# Exploring the role of electrostatic deposition on inhaled aerosols in alveolated microchannels

**DOI:** 10.1038/s41598-023-49946-w

**Published:** 2023-12-27

**Authors:** Ron Bessler, Saurabh Bhardwaj, Daniel Malka, Rami Fishler, Josué Sznitman

**Affiliations:** 1 Department of Biomedical Engineering, Technion–Israel Institute of Technology, Haifa, Israel; 2 Graduate Program in Nanoscience and Nanotechnology, RBNI, Technion–Israel Institute of Technology, Haifa, Israel; 3 Department of Applied Mechanics, Motilal Nehru National Institute of Technology Allahabad, Prayagraj, Uttar Pradesh India

**Keywords:** Biomedical engineering, Mechanical engineering

## Abstract

Large amounts of net electrical charge are known to accumulate on inhaled aerosols during their generation using commonly-available inhalers. This effect often leads to superfluous deposition in the extra-thoracic airways at the cost of more efficient inhalation therapy. Since the electrostatic force is inversely proportional to the square of the distance between an aerosol and the airway wall, its role has long been recognized as potentially significant in the deep lungs. Yet, with the complexity of exploring such phenomenon directly at the acinar scales, in vitro experiments have been largely limited to upper airways models. Here, we devise a microfluidic alveolated airway channel coated with conductive material to quantify in vitro the significance of electrostatic effects on inhaled aerosol deposition. Specifically, our aerosol exposure assays showcase inhaled spherical particles of 0.2, 0.5, and 1.1 μm that are recognized to reach the acinar regions, whereby deposition is typically attributed to the leading roles of diffusion and sedimentation. In our experiments, electrostatic effects are observed to largely prevent aerosols from depositing inside alveolar cavities. Rather, deposition is overwhelmingly biased along the inter-alveolar septal spaces, even when aerosols are charged with only a few elementary charges. Our observations give new insight into the role of electrostatics at the acinar scales and emphasize how charged particles under 2 µm may rapidly overshadow the traditionally accepted dominance of diffusion or sedimentation when considering aerosol deposition phenomena in the deep lungs.

## Introduction

Resolving the fundamental transport mechanisms of inhaled airborne particles is critical for inhalation therapy and efficient drug delivery to the lungs^1,2^. This is especially true for systemic and topical delivery to the deep respiratory regions (i.e. pulmonary acini) that comprise a vast surface area and  ~ 90% of total lung volume^3^. In assessing the governing aerosol deposition mechanisms at the sub-millimeter acinar length scales, particle dynamics have been traditionally characterized by two leading mechanisms, i.e. gravitational sedimentation and Brownian diffusion^4–7^. In practice inhalation therapy relies on pharmaceutical devices that accumulate high amounts of electrostatic charges on airborne particles^8^, created during dispersion from physical contact with the inhaler components or interactions with other particles^9^. Moreover, charged droplets can form spontaneously during the disruption process of the electrical double layer in the fluid as is the case in atomizer generators^9^. To date, however, how electrostatic mechanisms affect aerosol deposition at the smallest scales of the lungs has received comparatively little attention and is still not fully understood^8^.

The effect of electrical charge on inhaled particles has been recognized for nearly a century^10^. The electrostatic force, also known as Coulomb’s law, influences aerosol deposition by two concurrent mechanisms^11^. (i) The first draws on electrostatic repulsion that arises between charged particles in a dense aerosol cloud; a phenomenon known as *space charge* that is typically absent in inhalation therapy but may occur with high concentrations (> 10^12^ particles/m^3^) combined with charge levels on the order of hundreds of electronic charges^12^. (ii) The second mechanism is known as the *induced image charge*^13^ (see SM Fig. S1). There, a charged aerosol in proximity of the airway wall will cause the neutralized tissue surface to experience a dielectric effect (i.e. charged or dipole particles will reorient themselves by an external electric field) and will result in the creation of attractive forces between the airborne particle and the tissue surface. This latter mechanism is acknowledged to be more prevalent in inhalation therapy^11^ and can often lead, depending on particle size, to increased deposition in the extra-thoracic (e.g. mouth, throat) and upper respiratory airways^14–17^, when reasonably large charge levels exist (i.e. at least a few tens of electronic charges).

Early in vivo animal studies have supported the notion of electrostatic charge altering pulmonary deposition outcomes^18^, following which experiments in human subjects found aerosol deposition to be proportional both to charge and mechanical mobility *B*; a constant dependent on the carrier fluid (air) and the aerosol diameter *d*_*p*_^19^. These seminal efforts emphasized the coupled effect of charge with other deposition mechanisms (e.g., impaction, sedimentation, diffusion). However, such studies were limited to quantifying total deposition at the whole lung level, whereas the ability to characterize how electrostatic mechanisms influence local deposition patterns in different lung regions has remained largely absent. Since the electrostatic force is inversely proportional to the square of the distance between the charges (${F}_{elec}\propto {r}^{-2})$, the *induced image charge* is anticipated to become significant at the small airway scales, notably in the alveolar regions^19^. While the subject of some whole-lung simulations^20,21^, this hypothesis has never been directly explored in experiments.

To date, in vitro studies have been widely limited to upper respiratory tract models in quantifying overall deposition. For example, it was shown that even a single electron can play a significant role in affecting fine particle deposition in a simple trachea-like model^22^. Another study in a throat cast replica gave rise to up to a fourfold increase in deposition for 0.5–10 μm particles when the net charge was doubled^23^; a result in line with more recent computational fluid dynamics (CFD) simulations^24^. To the best of our knowledge, the smallest models explored in vitro have consisted of hollow casts of human airways extending down to the 6th generation^25^, where charged submicron particles showed a ~ 5 to sixfold deposition increase. One argument advanced on the prevalence of electrostatic effects in upper airways lies in the exposure of inhaled aerosols to high levels of relative humidity (> 90%) that would reduce their electrostatic charge^15^. Yet, the short time of flight along the inhalation route (~ 1 s) combined with the high electrostatic charge accumulated from the inhalers (~ 10^1^–10^2^ e) cannot yield significant charge decay over such narrow time scales^8,22,23,26,27^. Instead, the anticipated role of electrostatics at the smallest pulmonary length scales calls for renewed interest in deciphering the fate of inhaled aerosols in the acinar depths.

Motivated by these shortcomings, we explore in vitro the role of electrostatic forces on the transport and deposition of inhaled aerosols directly at the length scales of the pulmonary acinus. Leveraging state-of-the-art microfabrication techniques, we present an alveolated *airway-on-chip* that consists of an airway microchannel lined with anatomically-inspired alveolar cavities spaced by septal lining, entirely coated with electrically conductive material to realistically emulate the conducting properties of the parenchymal tissue. We quantify deposition patterns for a range of inhaled aerosol sizes acknowledged to access in vivo the acinar regions and traditionally considered to deposit under the leading influence of gravitational sedimentation or Brownian diffusion. By exploring two distinct electronic charge distributions, i.e. intrinsically charged atomized particles (i.e. > 10^2^ e) versus neutralized ones (i.e. Boltzmann distribution), we showcase how the existence of a non-zero charge can dominate the other deposition mechanisms and fundamentally alter local deposition outcomes; a phenomenon most prominent at the septal ridges in particular for highly-charged aerosols. Not only do our experiments give new insight into the role of electrostatics for aerosol transport at the sub-millimeter airway scales, these efforts also call for revisiting the underlying paradigms of acinar particle deposition in inhalation therapy.

## Materials and methods

### Alveolated channel design

**Figure 1:**
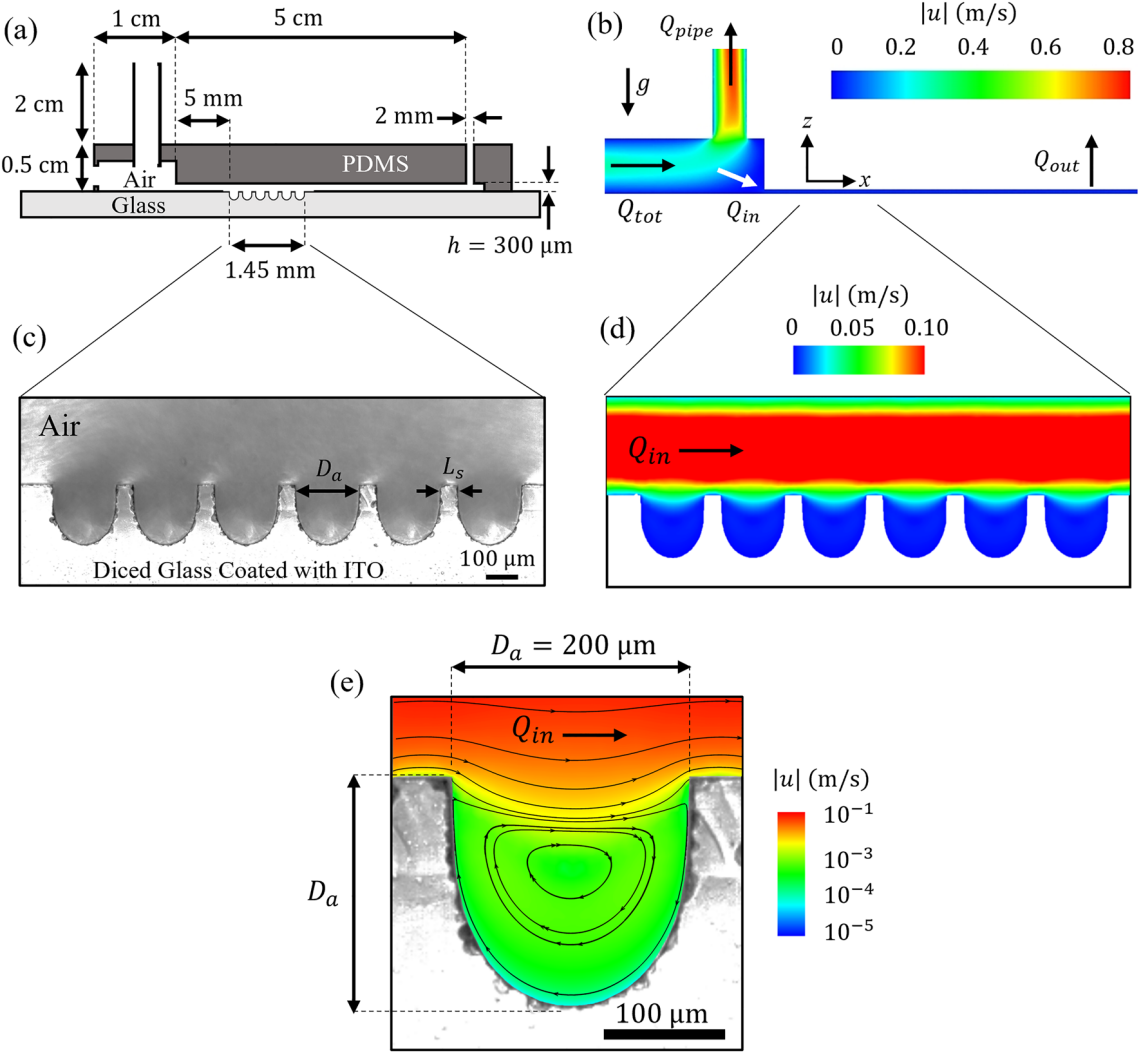
(**a**) Schematic of the alveolated channel model dimensions (not to scale). The model consists of a PDMS cover on top of glass coated with ITO. (**b**) Corresponding flow magnitudes obtained from Computational Fluid Dynamics (CFD). The total flow rate ${{\varvec{Q}}}_{{\varvec{t}}{\varvec{o}}{\varvec{t}}}$ enters the reservoir and splits into 2 flows: ${{\varvec{Q}}}_{{\varvec{p}}{\varvec{i}}{\varvec{p}}{\varvec{e}}}$ reduces the high velocities and ${{\varvec{Q}}}_{{\varvec{i}}{\varvec{n}}}$ which draws a slow stream of PSL aerosol-laden airflow through the leading channel. ${{\varvec{Q}}}_{{\varvec{i}}{\varvec{n}}}$ flows into the alveolated airway microchannel using a syringe pump connected to the output ${{\varvec{Q}}}_{{\varvec{o}}{\varvec{u}}{\varvec{t}}}$. (**c**) Bright field image (inverted microscope) of the alveolated region of the microchannel which features six alveoli. (**d**) Corresponding CFD simulations of the velocity magnitudes in the alveolated channel exhibiting characteristic Poiseuille flow within the main channel. (**e**) Velocity field and magnitude in a single alveolus exhibiting a separated flow with a slow recirculating vortex.

The proposed alveolated *airway-on-chip* design is inspired by seminal numerical^28–30^ and experimental studies^31,32^, including more recent microfluidic models of simple alveolated ducts^33–35^. The microfluidic device comprises a straight channel lined with *n*_*a*_ = 6 identical trenches mimicking the shape of 2D alveolar-like cavity structures. The cylindrical shape of the cavity along the spanwise cross-section reduces the flow to 2D and enables a clear view of the particles within^33^. The alveolar-like cavity is positioned in series along the bottom side of the channel with respect to gravity (Fig. 1a–c). Each alveolus is characterized by a diameter of *D*_*a*_ ~ 200 μm, in line with the range of anticipated alveolar sizes in average human adult lungs^36^. The alveolar cavities are spaced apart by a fixed distance (Fig. 1c); we refer here to this spacing as the *septal* distance (*L*_*s*_ = 50 μm), in analogy to the septal tissue that characterizes interalveolar structures along the acinar ducts^3^. We note that in our in vitro models the septal space is larger than true anatomical values found in situ^37^ (i.e. ~ 10–15 μm as evaluated from bronchoscopy in human adults^38^) and results from intrinsic limitations using microfabrication techniques (see details below) that cannot yield the same space-filling properties inherent of the pulmonary acinus^39^; a point also discussed in recent reviews^40^. The height of the rectangular channel is *h* = 300 μm and similar to the average diameter of acinar ducts^36^, whereas the width of the channel (*w* = 5 mm) is chosen to guarantee a quasi-two-dimensional (2D) flow along the streamwise direction (as *h* <  < *w*).

We mimic in experiments steady-state airflows that approximate characteristic physiological flow conditions under quiet inhalation^4,41^. Since our rationale lies in exploring the mechanistic determinants of deposition of charged aerosols traveling past the small bronchi of the conducting airways and entering the acinar regions, we specifically model aerosol deposition in proximal generations of the lung acinus, i.e. the *respiratory bronchioles* where alveoli first appear along the respiratory tract^36^. In the proximal alveolated ducts, the characteristic Reynolds number (Re) is anticipated to lie in the range of ~ 0.3–1.0^4,41^. With the present aerosol exposure setup (see below), we achieve a Re ~ 0.4 where Re $=Uh/\nu$; here, $\nu$ is the kinematic viscosity of air, $U$ is the characteristic mean airflow velocity in the channel resulting from the exposure flow rate ${Q}_{in}$. The total channel length is 5 cm, and the first alveolus is positioned 5 mm downstream from the microchannel inlet (Fig. 1a), corresponding to a distance more than two orders of magnitude larger than the anticipated entrance length *L*_*e*_ at such low Re (on the order of *L*_*e*_ ~ 0.05*h*Re). Such conditions give rise to fully-developed laminar Poiseuille flow profiles in the alveolated region of the channel (Fig. 1d; see simulations below). Note that our assumption of constant inhalation conditions is adequate as the Womersley number Wo, assessing the importance of flow unsteadiness in the deep lung regions is significantly smaller than unity under quiet breathing (Wo <  < 1). Hence, acinar ductal airflows may be considered to be a first approximation as quasi-steady^41,42^.

In analogy to the seminal efforts mentioned above, we emphasize that our acinar duct model features fixed walls that contrast with alveolar wall deformations resulting from intrinsic breathing motion. This limitation holds important ramifications as ensuing flow field topologies in the individual alveolar cavities of the microchannel are characterized by a single recirculating vortex (Fig. 1e), as previously established for geometries of similar aspect ratio (~ 1:1) at low Reynolds number^33,43,44^. In the absence of alveolar wall motions, the existence of a separated cavity flow prohibits any convective exchange between alveolar and ductal flows^45^. In turn, our experimental setup is designed to explore aerosol deposition respectively along the channel (i.e. septal space) and within the alveolar cavities as a result of three leading deposition mechanisms: electrostatic forces, gravitational sedimentation, and Brownian diffusion, or a combination thereof.

### Microfabrication

To fabricate the quasi 2D alveolar-like cavity structures, we use an Automatic Dicer (DAD3350, Disco) with a Disco diamond blade (MBT-B433-SD600L25MT38DD, Disco Japan) applied to transparent microscope glass slides (Marienfeld, dimensions: 76 × 26 × 1 mm^3^). Six straight identical cuts are applied along the spanwise (*y*-axis) of the glass slide, leading to cavity-like indents in the glass (see Fig. 1c). The cutting speed was set to 2 cm/s, and the spindle revolution was fixed at 35,000 RPM. The dicing depth was set to *D* = 200 μm with a blade thickness of *t* = 200 μm to a produced aspect ratio for the cavity of 1:1. The spacing step between each cut was set to 250 μm, leading to a final 50 μm separation between each cavity (Fig. 1c). Afterward, the diced glasses went through a cleaning process first using an air jet followed by washing with DI water, followed by 10 min immersion in an acetone container, and eventually washed with 2-propanol. Glasses were then dried under a jet of nitrogen gas. The dimensions of the alveolar-like cavities and spacing were finally measured using a bright-field mode on an inverted microscope (Nikon Eclipse Ti).

The microchannel top enclosure, consisting of three of the channel walls (i.e. top and sides), was created by polydimethylsiloxane (PDMS) casting. The PDMS was mixed with a curing agent (Dow Corning) at a 10:1 weight ratio and poured on a template. The negative template of the top channel was custom-designed by SolidWorks and was 3D printed using a Prusa SL1 printer (3DM-ABS Advanced Material). The layer height of the printing was set to 0.025 mm and the exposure time to 10 s. After cleaning the model template with isopropyl alcohol, we strengthen the template by curing it for 60 min at 60 °C (Formlabs, Form Cure FH-CU-01). To protect the template from deformation, the PDMS was peeled after 24h resting within the mold under ambient conditions instead of using the oven. Only from the second time of pealing the PDMS would be pealed smoothly from the templated. To create an outlet port, we punched the model using a biopsy punch of 2mm size (Miltex, 3331). The undesired outlets resulting from the dicing of the cavities were sealed using Plasticine. Using a high-frequency generator (ETP BD-10ASV) we adhered the PDMS cover to the diced glass and baked the model for 2h at 65 °C. Afterward, the PDMS-glass interface was coated with an additional layer of PDMS to strengthen the adherence and verify sealing. The final layer is dried for an additional 15 min at 65 °C.

### Conductive layering of the alveolated microchannel

Prior to the PDMS bonding step, the glass slide containing the bottom alveolated surface is covered with a thin layer of indium tin oxide (ITO) using Plasma Enhanced Atomic Layer Deposition (Ultratech, Cambridge Nanotech Fiji G2). This supplies a conductive layer to the microchannel without compromising the transparency of the glass. The deposition process is limited to the microchannel region while the rest of the glass is protected by Kapton tape to prevent ITO deposition on the rest of the glass regions to be adhered with the top PDMS channel cover (see next section).

Briefly, Ti-doped indium oxide films are deposited using super cycles of indium oxide and titanium oxide. At the reactor, the temperature was set to 220 °C using Trimethylindium (TMI, 99% In) as an In metalorganic precursor, Tetrakis Dimethylamino Titanium (TDMAT, 99%) as a Ti metalorganic precursor and ${O}_{2}$ Plasma as an oxidizer, respectively. We conducted a cycle ratio of 30:1 (In to Ti). High-purity Ar was used as a carrier gas for both half-cycles. The TMI precursor was kept at room temperature (RT), while the TDMAT precursor was heated to 75 °C. Deposition took place following  ~ 250 cycles, which yielded a total ITO film thickness of  ~ 30 nm. Using a Rudolph AutoEL-II ellipsometer, the deposited film refractive index was measured to 2.07 at a 632 nm wavelength.

It is noteworthy to recall that the electrical conductivity of general lung parenchymal tissue closely approximates that of saline water with ${{\sigma }_{_{\text{H}_{2} \text{O}}}}\!\!\sim \!\!1$ S/m^22^. Hence, such large value is sufficient for electrical charges within lung tissue to exhibit remarkably rapid time scales, and thus facilitating the application of *induced image charge* phenomena^19^. To address the inherently low conductivity of the glass substrate ($${\sigma }_{{\text{glass}}}\sim$$ 10^–11^ S/m), we introduced an indium tin oxide (ITO) layer atop the glass channel; this additional layer substantially enhances the conductivity to $${\sigma }_{{_\text{ITO}}}$$~10^3^ S/m, as verified through four-point-probe (FPP) measurements. This elevated conductivity level enables the manifestation of *induced image charge* phenomena and replicates closely the anticipated electrostatic effects in situ within the lungs. A more comprehensive discussion of the electrical time scale analysis is available in the Supplementary Materials (SM).

### Aerosol exposure setup

In line with recent aerosol exposure assays in *airway-on-chip* models^46,47^, we use fluorescent polystyrene latex (PSL) particles (Fluoromax red and green fluorescent microsphere 1% solid, Thermo Scientific). Most relevant to the present study, PSL tends to inherently accumulate an excess amount of electrostatic charge during the aerosolization process^48^. Hence, in an effort to explore the role of electrostatic on deposition in our models, relative to the other well-established deposition mechanisms in the deep lungs (i.e. diffusion, sedimentation), we select three distinct monodisperse aerosol sizes that are known to reach the acinar regions; namely, *d*_*p*_ = 0.2, 0.5 and 1.1 μm (*ρ*_*p*_ = 1050 kg/m^3^). Such sizes underline respectively particle deposition under the leading influence of Brownian diffusion (*d*_*p*_ = 0.2 μm) and gravitational sedimentation (*d*_*p*_ > 1 μm), whereas 0.5 μm is acknowledged as lying within the small size window of inhaled aerosol sizes dominated by convection that are mostly exhaled under normal breathing and only deposit at low quantities due to weak diffusional or weak gravitational forces^4,5^.

The PSL particles are diluted in DI water to a high concentration (~ ${10}^{9}$particles/ml) to achieve sufficient deposition data for ensemble statistics over relatively short exposure times^4^ (i.e. ~ 10 to 20 min depending on particle size). Nonetheless, it was crucial to prevent the formation of aerosol aggregates due to the high concentration of suspended PSL particles, as previously described in detail^5^. To address this concern, prior to the experiment the microspheres underwent a 30 min sonication in an ultrasonic water bath (Elma Elmasonic S10). The final weight percentage of the particle suspension was 5%; this concentration was carefully selected to ensure the generation of an aerosol with less than 1% aggregates, assuming that the water droplets exit the atomizer with specified parameters (i.e. a count mean diameter of 0.35 μm and a geometrical standard deviation of 2, as provided by the manufacturer). Our approach aligns well with the analysis presented in previous studies by^4,5^, based on the assumption of a log-normal distribution of droplet sizes and monodisperse spherical particles. During the experiments, a magnetic stirrer is used throughout to prevent particle sedimentation at the bottom of the container.

**Figure 2:**
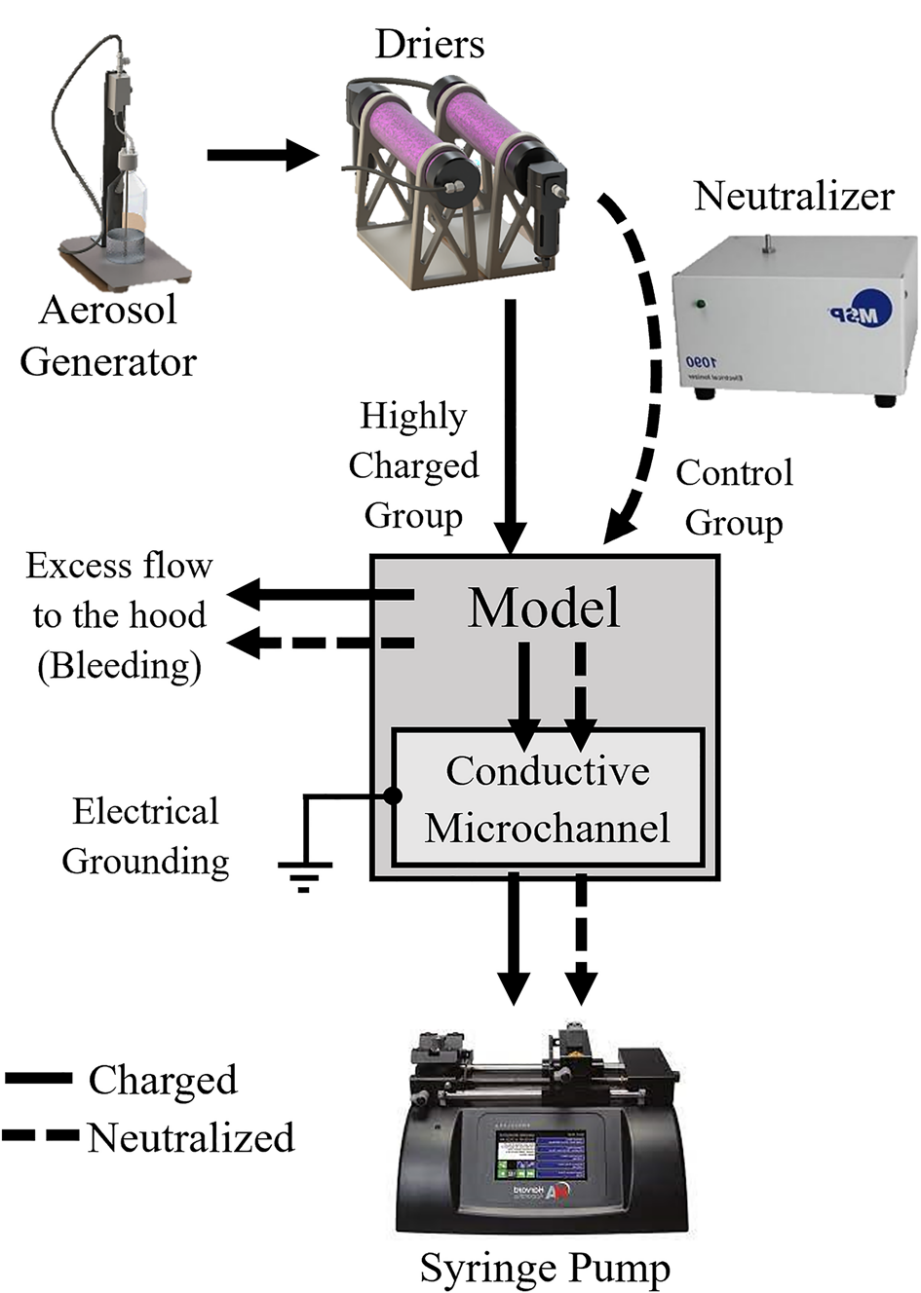
Schematic illustration of the experimental aerosol exposure setup, generated using Microsoft PowerPoint, highlighting the steps from aerosol generation to deposition inside the model (see Fig. 1). Two experimental groups are independently explored: (i) particles that are intrinsically highly-charged during their generation via the atomizer (full line) and bypass the neutralizer; and (ii) aerosols with a neutralized charge (dashed line) corresponding to the Boltzmann charge distribution (see SM Fig. S2).

The aerosol exposure setup (Fig. 2) consists of an aerosol generator (Model 3076, TSI) that uses a collision-type atomizer with filtered air as the gas source with a pressure set to 2 bar. To remove water vapor from the aerosols under minimal loss, aerosols are first passed through two consecutive diffusion driers (Model 3062, TSI). An antistatic tube is used to feed the aerosol-laden flow with a nominal flow rate of ${Q}_{tot}=$ 1 L/min measured using a flow meter (Model 4100, TSI). Before the entrance to the microchannel inlet, the aerosol-laden stream is split using a chamber with a bleeding chimney located at the chamber top (with outflow ${Q}_{pipe}$). The stream toward the microchannel inlet is further reduced by strategically locating the inlet at the corner of the chamber feeding the microchannel (Fig. 1b) where airflow velocities are lowest following numerical simulations. To deliver a physiologically-relevant flow rate locally within the alveolated duct model (see earlier discussion), a stream of air is pulled into the microchannel using a syringe pump (PHD Ultra, Harvard Apparatus) connected to the channel’s outlet. The syringe pump is set to ${Q}_{in}$= 2 mL/min resulting in a steady-state, laminar flow rate at the microchannel entrance mimicking acinar flows under quiet breathing conditions (Re ~ 0.6). To guarantee an identical electrical setup prior to each exposure assay and to eliminate the potential effects of excess surface charge, we electrically ground the coated conductive glass via a custom external connector wired to the inner surface of the microfluidic channel (Fig. 2).

Aerosolized PSL particles are intrinsically charged during the aerosolization process due to random fluctuations in the concentrations of ions in the liquid. We infer the expected charge of the charge PSL particle in our exposure setup to be approximately > 10^2^ e based on previous experiments^48,49^. As such, these aerosols represent our “charged” group. Concurrently, as a control group we compare the same aerosolized particle sizes under neutralized conditions using an electrical ionizer (1090 MSP, TSI); we refer to such aerosols as belong to the “neutralized” group. The neutralizer operates most effectively on dry particles and is thus sequentially placed after the diffusion dryers (Fig. 2). Briefly, the neutralizer produces a high electric field around an electrode that creates high concertation packets of positive and negative ions. The input aerosol stream is exposed to a high concentration of bipolar ions that neutralizes the aerosols and results in aerosols with an equilibrium charge distribution (i.e., Boltzmann charge distribution; see SM Fig. S2). We emphasize that the “neutralized” aerosol group is not characterized by zero charges; rather, the neutralizer reduces the aerosol charge down to just several electrons (e), resulting in a decrease of over two orders of magnitude compared to aerosols under charged conditions. The average unit of charge $\overline{n }$ of each population is in turn 1.06 e, 1.67 e, and 2.48 e for ${d}_{p}=$ 0.2 μm, 0.5 μm, and 1.1 μm, respectively (see SM Fig. S2).

### Computational fluid dynamic (CFD) simulations

The steady laminar flow of the continuous phase (i.e. air with ${\rho }_{f}=1.225 {\text{kg}}/{{\text{m}}}^{3}$ and ${\mu }_{f}=1.7894\times {10}^{-5} \mathrm{Pa\cdot s}$) is governed by the mass and momentum conservation equations (i.e., Navier–Stokes equations) and numerically solved using the finite volume method (FVM) using a commercial solver (i.e., Fluent 19.3, ANSYS, Inc). The momentum equations are discretized using a second-order upwind scheme for velocity and a second-order scheme for pressure, whereas, for coupling the velocity and pressure fields, the SIMPLE algorithm is applied along with a least-squares-based scheme for gradients. A numerical alveolated duct model identical to that used in the experiments (Fig. 1) was meshed with tetrahedral cells (~ 4.8M) using commercial meshing software (ANSYS ICEM) and converted to polyhedral mesh (~ 2.1M) in Fluent following a rigorous convergence study. For numerical modeling purposes, the model inlet is treated as a steady and uniform velocity inlet with a flow rate equal to ${Q}_{in}$=2 mL/min (as described above) and zero pressure applied at the outlet.

### Deposition quantification

**Figure 3:**
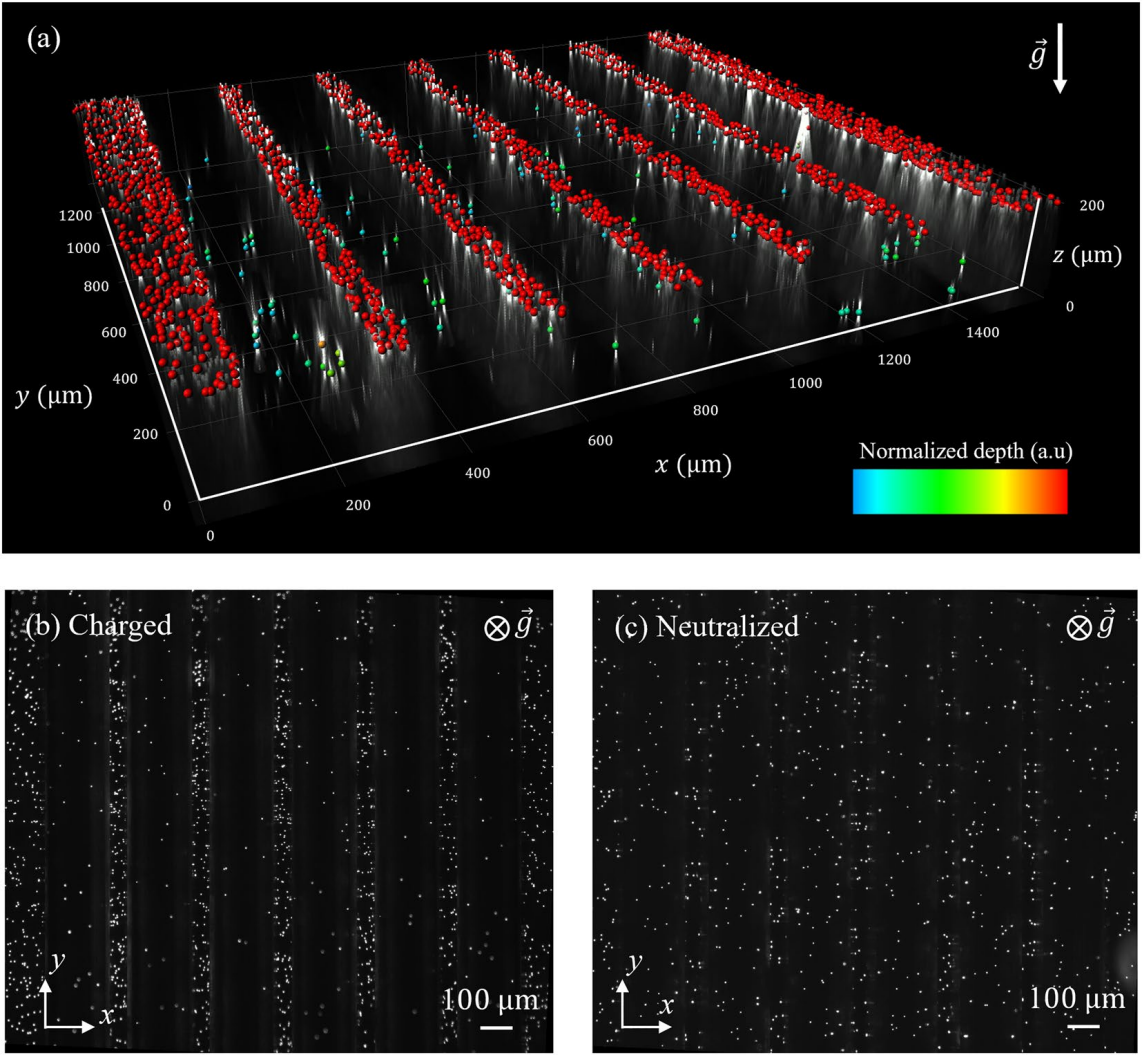
Microscopy imaging in the alveolated airway microchannel following deposition assays. (**a**) Reconstructed 3D visualization of the alveolated section of the microchannel (refer to SM Video 1) created using IMARIS. In this visualization, each colored point represents a deposited charged PSL particle with *d*_*p*_ = 0.5 μm (as detailed in the Methods section). (**b**) and (**c**) provide a qualitative comparision for deposition patterns presented from the top view via an inverted microscope. The particles presented are PSL particles with *d*_*p*_ = 1.1 μm. (**b**) depicts the charged case, while (**c**) represents the neutralized control group. The data in both (**b**) and (**c**) are extracted and analyzed using image-based local maxima analysis through ImageJ (see Methods).

Following each aerosol exposure experiment, alveolated channels were thoroughly examined using an inverted fluorescent microscope (Nikon Eclipse Ti) at varying magnifications (40X, 20X and 10X) depending on particle size (i.e., 0.2, 0.5 and 1.1 μm, respectively). The quantification of deposition involved identifying the precise planar locations (*x*, *y*) of individual deposited fluorescent particles through digital processing using local intensity maxima in ImageJ software (presented below in the Results section; e.g. see Fig. 3). This approach allowed us to determine the spatial distribution of particle deposition within the in vitro model. Furthermore, we distinguish between two deposition groups as presented below: “septal deposition” (i.e. particles located along the septal zones) and “cavity deposition” representing deposited aerosols lining the alveolar cavities.

### Statistical analysis

For each particle size, we conducted n = 3–7 independent experiments for each aerosol size and group (i.e. charged vs. neutralized). A two-way analysis of variance (ANOVA) was used to examine the effect of the experiment settings. Our two independent variables were (i) the aerosol size (i.e., ${d}_{p}\in \left\{\mathrm{0.2,0.5,1.1}\right\} \mathrm{\mu m})$ and the electrostatic charge group (i.e. q $\in \left\{{\text{neutralized}},\mathrm{ charged}\right\}$). The dependent variable was the density ratio of septal to alveolar deposition (${C}_{s}/{C}_{a}$) as further discussed below in the Results and Discussion section.

## Results and discussion

To first give some insight into typical deposition data we present a reconstructed 3D visualization in Fig. 3a for *d*_*p*_ = 0.5 μm, in the case of charged aerosols (see SM Video 1). Figure 3b and c demonstrate the substantial impact of the charge on 1.1 μm particle deposition. Figure 3c presents how the influence of gravitational forces gives a homogeneous deposition pattern (observing from a top view). In contrast, when charge is introduced (as seen in Fig. 3b), a distinct separation emerges. As a first observation, we note that deposited particles are predominantly located along the septal spacings (*L*_*s*_) rather than in the alveolar cavities. This immediately points to a highly localized concentration of deposited aerosols; a point we quantitively explore below. It is crucial to emphasize that our model's cavity orientation aligns with the direction of gravitational sedimentation along the $-\widehat{z}$ direction. In turn, any deviation from this orientation would result in even lower cavity deposition. Furthermore, this orientation significantly impacts the deposition of larger 1.1 μm particles and, to a lesser extent, 0.2 μm particles.

### Deposition fractions along the alveolated channel

**Figure 4:**
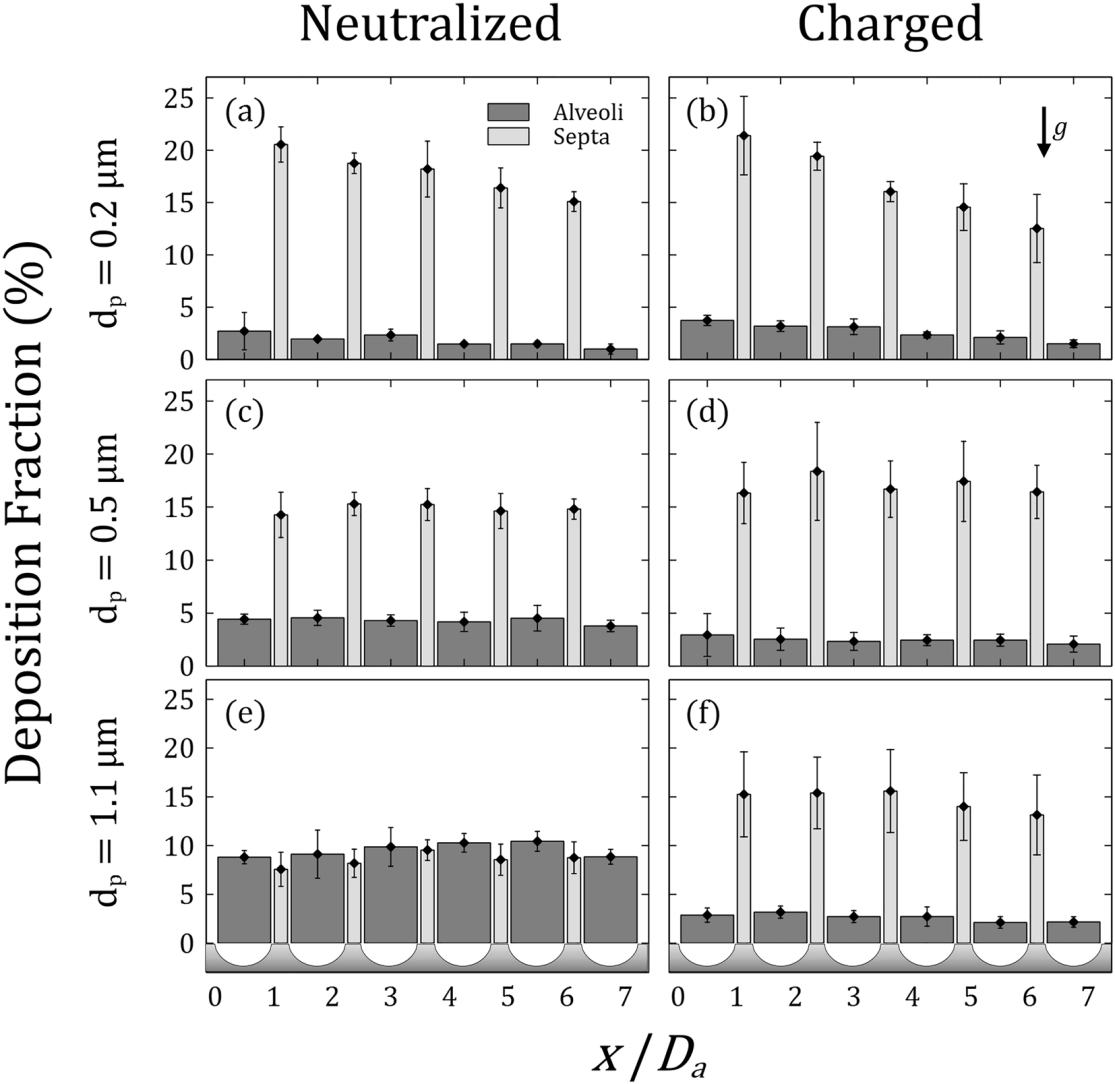
Summary of the deposition fractions (DF) for aerosol exposure assays in the alveolated airway microchannels. Deposition fractions are presented for each alveolus and septum number along the *x* direction of the in vitro model (see Fig. 1). Data are shown according to the three distinct particle sizes ${d}_{p}$ (rows) and columns discriminate between exposure to neutralized or charged aerosols. Dark gray represents deposition within the cavities and light gray deposition on the septa, respectively. Note that the schematic drawing below the graphs in (**e**) and (**f**) represent the alveoli and septa positions along the *x* direction for improved spatial orientation.

We extract from microscopy imaging the deposition fractions (DF) for each alveolus and septum along the streamwise axial position of the alveolated channel (*x/D*_*a*_) after completion of each exposure assay. A summary of the results is presented in Fig. 4, where we present deposition according to particle size (Fig. 4, rows) and group i.e. neutralized versus charged (Fig. 4, columns). The histograms are normalized according to the total number of deposited particles. We discriminate between deposition along the septal spaces (i.e., light gray histograms) and within the alveolar cavities (i.e., dark gray histograms); this distinction is particularly relevant when considering the fate of inhaled aerosols for alveolar deposition (see discussion below). We immediately note that deposition is foremost biased to septal spacings and most striking when considering 0.5 and 1.1 μm aerosols in the “charged” group. In general, septal spacings each carry a DF of around 15% while individual cavities hold a DF typically  < 5%.

For the case of 0.5 μm aerosols (Fig. 4c,d), the difference in the distribution of DF along the alveolated channel appears rather negligible between the neutralized and charged cases, in the absence of significant diffusion or sedimentation for this particle size^5,50^. Yet, in the charged case (Fig. 4d) deposition is slightly higher along the septal spaces at the cost of further reducing deposition within the individual cavities. This latter observation hints to the increasingly dominant role of electrostatic forces when particles are charged. Indeed, we recall that our PSL particles are anticipated to accumulate charges of several hundred electrons^48^; an amount that is comparable to commonly-available inhalation devices such as dry powder inhalers (DPI)^51^ and metered dose inhalers (MDI)^12^.

We now turn our attention to the most discernable case differing from the general trend, i.e., neutralized particles with *d*_*p*_ = 1.1 μm (Fig. 4e). This latter result may be grasped by recognizing that the relative role of sedimentation becomes more important as velocity magnitudes of airflows decrease in the acinar depths (see definition of the gravity number below)^4^. In the absence of convective exchange between the main channel and the recirculating cavity flow (Fig. 1e), sedimentation along the direction of gravity increases the opportunity for aerosols to cross into the alveolar cavities and deposit without being conveyed back into the central channel^52,53^; a latter phenomenon witnessed during exhalation maneuvers in microfluidic in vitro models^47^. Over the short streamwise distance interrogated ($\le$ 1.45 mm), the axial length is not however sufficiently long to observe a negative gradient in the deposition that would result from gradual sedimentation as seen in larger in vitro acinar trees^54,55^ and small bronchial airway models^46^. Nevertheless, the influence of sedimentation becomes again largely obscured when particles are charged (Fig. 4f).

Upon considering the smallest particle size *d*_*p*_ = 0.2 μm (Fig. 4a,b), we observe a monotonic decrease in DF along the alveolated channel; a trend most noticeable for the case of charged aerosols along the septal spacings (i.e. DF spans > 20% passed the first alveolus down to < 15% when reaching the last), but also present in the alveolar cavities (Fig. 4b). Our experiments for 0.2 μm underline the superposition of Brownian motion on electrostatic forces and are evocative of *diffusional screening* occurring in the pulmonary acinus; a well-known physical phenomenon whereby particles that diffuse near an irregular surface are unlikely to reach more distal regions of the surface as they first collide with the nearest surface thereby acting as a “screen” behind which less accessible regions are hidden^56–58^. Here, we witness the streamwise depletion of deposited particles along the alveolated channel; a trend anticipated to be even more pronounced in larger acinar tree networks^58^.

### Septal versus alveolar deposition

**Figure 5:**
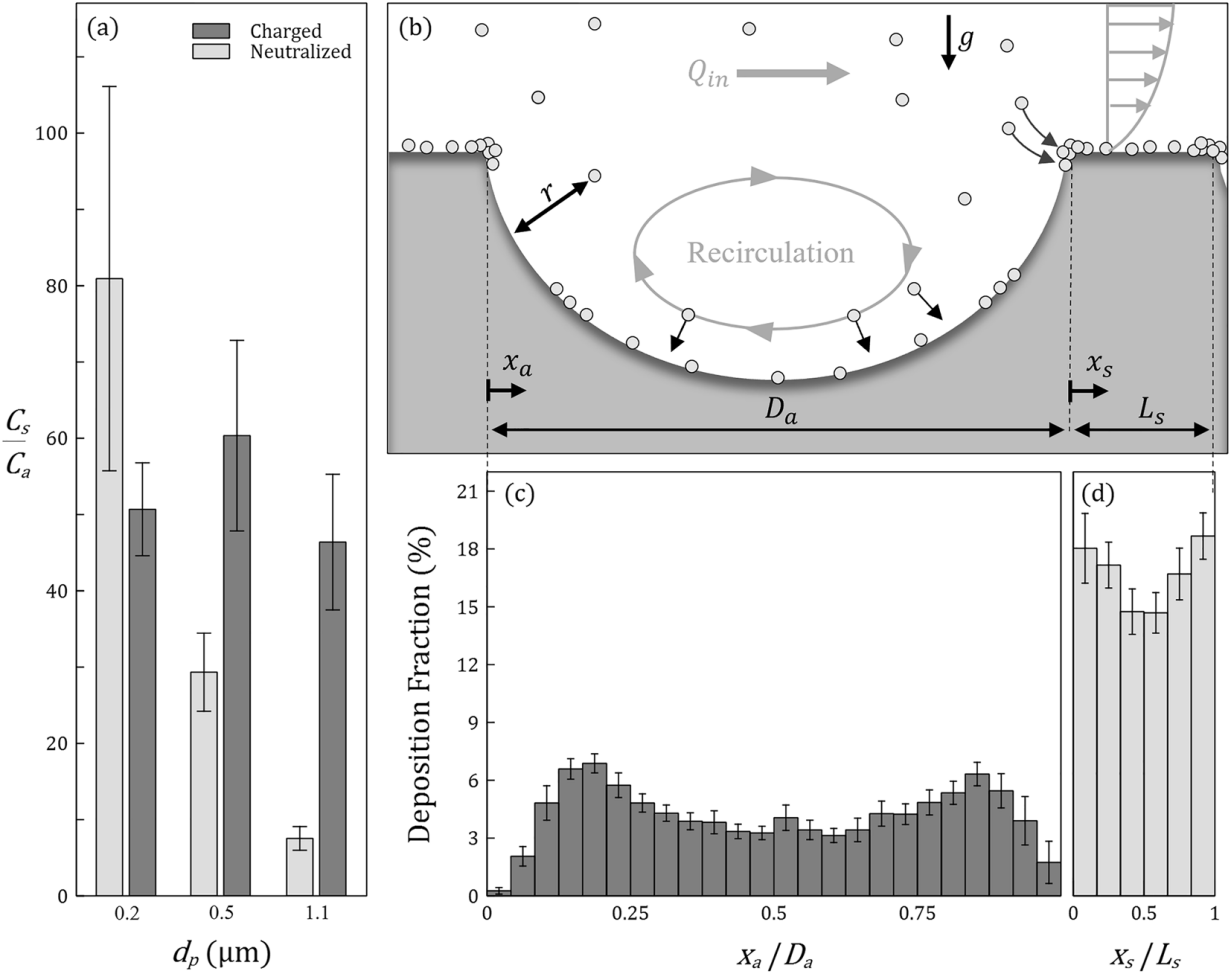
Comparison between local septal and alveolar deposition. (**a**) Histograms of the septal to alveolar particle deposition fraction density ratio ${C}_{s}/{C}_{a}$ as a function of particle size ${d}_{p}$ and charge (neutralized vs. highly-charged). (**b**) Schematic drawing (not to scale) showcasing deposition phenomena within alveoli and adjacent septal spaces. The main channel is characterized by Poiseuille flow with a flow rate ${Q}_{in}$. The majority of aerosols are deposited along the septal spaces, in particular near the septal ridges, rather than within the alveolar cavity. Histograms summarizing local deposition fractions (**c**) within the cavity and (**d**) along the adjacent septal spaces. Data are shown for the case of charged particles with ${d}_{p}=0.5 \mathrm{\mu m}$. Horizontal axes in (**c**) and (**d**) represent a normalized axial distance, where each bin equals a distance of 8.3 μm.

Motivated by the importance of targeting the acinar regions for inhalation therapy including systemic delivery^59–61^, we further explore differences between septal deposition (DF_*s*_) and alveolar deposition (DF_*a*_) and are specifically interested in evaluating the ratio DF_*s*_/DF_*a*_. We note that DF_*s*_ and DF_*a*_ depend on the areas *A*_*s*_ and* A*_*a*_ corresponding to the total septal and (concave) alveolar surface areas in our microchannel, respectively. Here, the ratio of total septal to alveolar surface area *A*_*s*_/*A*_*a*_ is a mere ~ 0.1 (see SM calculations and Fig. S3). In true lungs, however, *A*_*s*_/*A*_*a*_ is expected to be much smaller as our canonical model features septal spacings (*L*_*s*_) that are ~ 4 times thicker than in situ^38^. Motivated by in vivo deposition phenomena and in an effort to account for the manufacturing limitations of large ${L}_{s}$ values, we extract instead a particle deposition density (or concentration) defined as *C* = (DF)/*A*; we evaluate *C*_*s*_ and* C*_*a*_ denoting the septal and alveolar deposition densities, respectively. The ratio *C*_*s*_/*C*_*a*_ thus represents the relative importance of septal deposition where results are shown in Fig. 5a for all exposure assays. Values span nearly an order of magnitude (< 10 to > 80), underscoring accrued deposition along the septa regardless of particle size or charge. Such observation is in line with the general propensity for aerosols to deposit preferentially on septal ridges of the alveolar openings, as observed in computational models^5^, in vitro experiments^47^ and animal studies^62^.

It is important to emphasize that the present in vitro results pertain only to models with static walls that omit breathing dynamics and motion; this follows from technical constraints related to the fabrication techniques employed. Consequently, the absence of alveolar wall motion hinders convective exchange between alveolar and ductal flows, a well-known phenomenon from previous studies^45^. Therefore, the observed lower deposition within the cavities compared to the septal regions should not be surprising. However, even with the inclusion of moving walls, we would anticipate only a marginal increase in particle deposition within the cavities, consistent with findings from previous in vitro studies and numerical simulations^47^. Nevertheless, this study marks an initial phase, establishing the groundwork for exploring the role of electrostatic forces within the acinar region. However, the exact deposition patterns within real alveolar cavities in situ remain an open question. The present work represents an important stepping stone in ultimately addressing such question.

Until now, few studies have made any direct allusion to the significance of electrostatic effects on acinar deposition. Our experiments point to their importance towards deposition along the alveolar ridges when charge is finite as a result of the aerosolization process (Fig. 5a, dark gray histograms). From our analysis, the density ratio *C*_*s*_/*C*_*a*_ is dependent on charge (*p* = 0.0205 for $$q$$), diameter (*p* < 0.001 for *d*_*p*_), and the interaction between them (*p* < 0.001 for $$q{d}_{p})$$. Following the significant interaction, by looking at the simple main effects (SME) for the “charged” group, we note that $${d}_{p}$$ does not significantly affect the ratio *C*_*s*_/*C*_*a*_ (*p* = 0.2187); this corresponds in our experiments to the case when the density ratio remains nearly constant (*C*_*s*_/*C*_*a*_ ~ 50) regardless of particle size, as diffusion and sedimentation are entirely overshadowed. More generally, our findings also support the few seminal in vivo inhalation studies of charged aerosols in human volunteers that concluded on deposition enhancement arising from electrostatic charge^19^. Notably, the authors hypothesized that the primary site for such deposition enhancement would lie in the acinar regions for similar aerosol sizes inhaled in their experiments (i.e. 0.3, 0.6, and 1.0 μm).

Lastly, when the aerosol charge level is “neutralized” (Fig. 5a, light gray histograms), the SME analysis shows that ${d}_{p}$ is significant with regards to the density ratio (*p* < 0.0001). In turn, *C*_*s*_/*C*_*a*_ is strongly dependent on particle size underscoring then the relative importance of diffusion and gravity. The SME analysis for each particle size results in a significant effect of the charge level on the density ratio (i.e. *p* = 0.0035, 0.0008, 0.0003 for 0.2, 0.5 and 1.1 µm respectively). For neutralized 1.1 μm particles, sedimentation favors more deposition in the alveolar cavities compared to 0.5 and 0.2 μm particles, given the direction of gravity^63^. While *C*_*s*_/*C*_*a*_ is then at its lowest, the septal deposition density is still > 7 times that within the alveoli. This outcome also emphasizes the mechanistic challenges of aerosol targeting inside alveolar cavities for efficient inhalation therapy^50^. In contrast, for neutralized 0.2 μm aerosols, the ratio *C*_*s*_/*C*_*a*_ significantly decreases when Brownian motion is superimposed onto electrostatic effects. Not only does diffusional screening operate within the main channel (as discussed above), aerosols that cross within the alveolar cavities still have some probability to travel back out when they are neutralized as a result of stochastic motion that contrasts with deterministic trajectories for sedimentation^5,47^.

### Role of electrostatic charge on local spatial deposition

At the acinar scales electrostatic forces emanating from the induced charge on the airway’s lumen are anticipated to become more prevalent as the distance *r* between an aerosol and the closest airway decreases (Fig. 5b). Recalling that the magnitude of the attraction force on such aerosol scales with |*F*_*elec*_*|*~ *(q*/*r)*^2^ (Coulomb’s law), we anticipate that as *r* becomes small, the electrostatic attraction force will prevent aerosols from escaping local deposition regardless of their size. To better characterize the mechanistic underpinnings of electrostatic forces towards such localized deposition phenomena, we quantify the spatial distribution of deposited aerosols within the cavities and along the septal spaces (Fig. 5c,d), respectively. For brevity, we limit results to *d*_*p*_ = 0.5 μm as our preferred candidate to isolate the role of electrostatic forces in airborne experiments. For the other sizes of particle distribution within the cavities see SM Fig. S4.

In Fig. 5c, we present deposition results superimposed for all six alveoli. Here, DF is shown as histograms of identical bin widths (i.e., *D*_*a*_/24 = 8.3 μm) along the non-dimensional distance (*x*_*a*_*/D*_*a*_*)* measured from the edge of the alveolus along the streamwise direction (see Fig. 5b). The resulting distribution exhibits a nearly symmetrical shape with respect to the cavity midpoint (*x*_*a*_/*D*_*a*_ = 0.5) with two symmetrical peaks. Since the horizontal distance *x*_*a*_*/D*_*a*_ represents a projected view of the alveolus’ parabolic curvature, this signifies that the sides of the cavity exhibit more surface area available for particle–wall interactions. However, as one approaches the vicinity of the septal edges (i.e., *x*_*a*_/*D*_*a*_ $\to \{\mathrm{0,1}\}$), we witness a nearly complete depletion in deposition despite the most surface area accessible for particle–wall interactions. This latter result can be better understood by examining DF on the ridges of the actual septal space (Fig. 5d), where the histograms are comparatively shown for the same physical bin width (i.e., *D*_*a*_/24 = *L*_*s*_/8 = 8.3 μm) along the non-dimensional distance (*x*_*s*_/*L*_*s*_) measured from the edge of the septum (see Fig. 5b).

Not only does Fig. 5d reveal particles’ affinity towards the septal space (compare DF in Fig. 5c and d), we also observe local peaks in DF located at each septal ridge (i.e. *x*_*s*_/*L*_*s*_ $\to \{\mathrm{0,1}\}$), with the local DF minimum positioned at the septal midpoint (*x*_*s*_ = *L*_*s*_/2). These variations in deposition can indeed be associated with flow recirculation regions, where the local flow is directed towards the wall with higher velocity, resulting in enhanced localized deposition. Additionally, it is possible that the septa might act as zones of high electrostatic attraction for two reasons: (i) compared with alveolar cavities, septa are the closest surface to interact with aerosols convected in the main channel, thereby enriching them with additional deposition. (ii) In line with fundamental electromagnetism^64^, the ridges represent two sharply curved edges (Fig. 5b) which we speculate may lead to concentrated electric fields $\overrightarrow{E}$ compared with the weaker electric field produced by the cavity's flatter walls. Recalling that ${\overrightarrow{F}}_{elec}=q\overrightarrow{E}$, such local concentrated electric fields could create larger attractive forces on nearby aerosols. While beyond the scope of the present article, these results open the door for future investigations.

### Non-dimensional considerations

As a final step, we revisit the governing mechanisms of aerosol deposition in our model with dimensional analysis. In the absence of inertial impaction^65^, aerosol deposition in the acinar depths has been traditionally acknowledged as resulting from sedimentation or Brownian diffusion depending on particle size. Hence, the following dimensionless parameters are typically introduced to characterize their respective roles^4,7,31,43^: the gravity number ${\text{H}}={\tau }_{p}{\text{g}}/{U}_{c}$ and the inverse particle Peclet number Pe^-1^ = *D*_*diff*_ /*U*_*c*_*L*_*c*_, where *D*_*diff*_ is the Stokes–Einstein diffusion coefficient. We recall that Pe^-1^ and H may be derived from conservation of linear momentum for an airborne particle (see SM). Yet, such mechanistic treatments implicitly assume ideally neutral aerosols (*q* = 0). Instead, the Coulomb force arising from particle–wall interactions should be accounted for, thereby leading to an additional group^22^, i.e., ${\text{Inc}}={C}_{c}{q}^{2}/3{\pi }^{2}{\mu }_{f}{d}_{p}16{\varepsilon }_{0}{{L}_{c}^{2} U}_{c}$, where *C*_*c*_ is the Cunningham correction factor (see SM).

**Figure 6:**
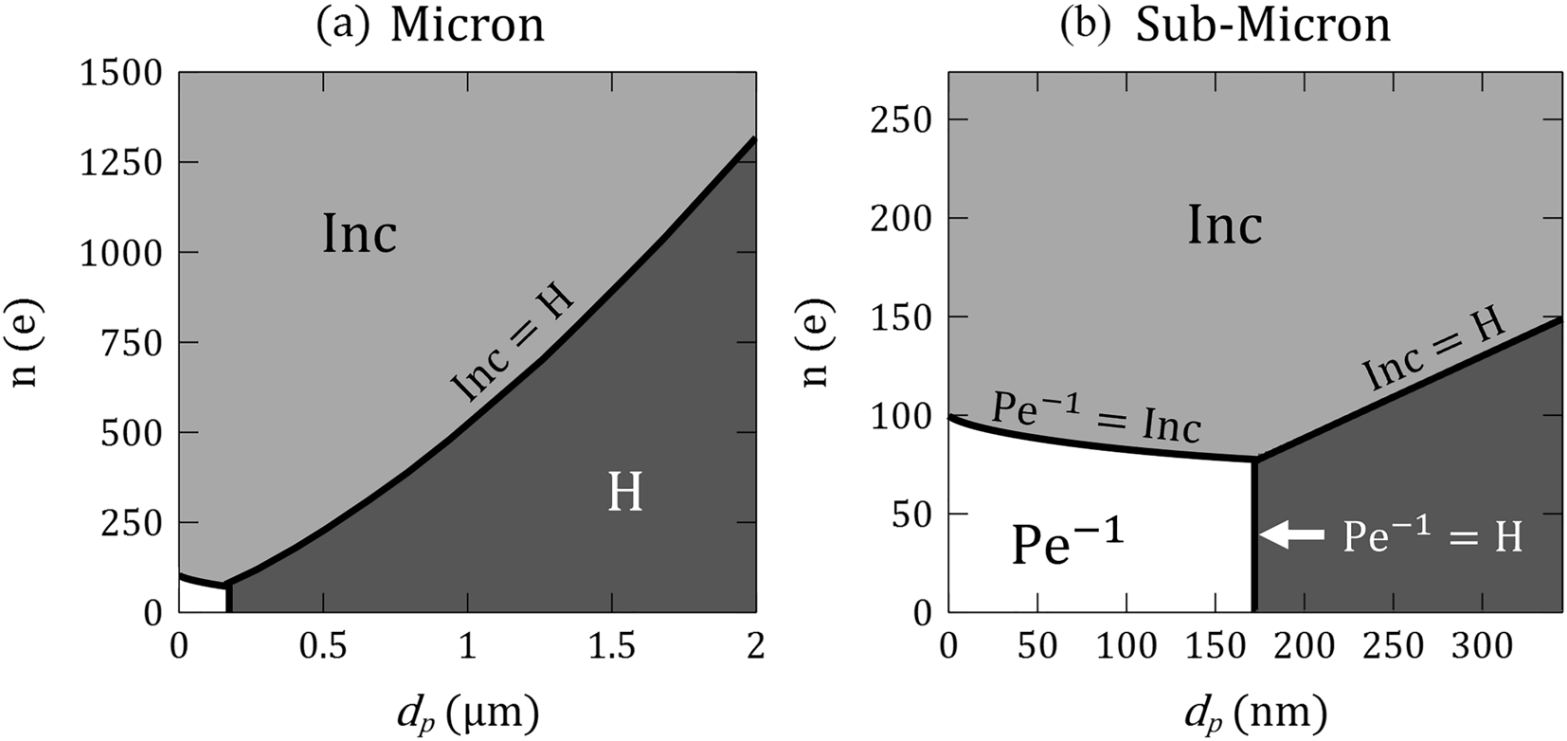
Phase map summarizing the leading dimensionless numbers (H, Pe^-1^ and Inc) as a function of particle diameter *d*_*p*_ (horizontal axis) and units of elementary charge e (vertical axis). The distinctive zones in the phase map underline areas where one group dominates over the others while the delineation between zones identifies where two groups are equal in an order of magnitude sense. (**a**) and (**b**) display different resolutions of the phase map. Note that the analysis is conducted for a characteristic velocity magnitude $U=3 {\text{mm}}/{\text{s}}$ and a characteristic length ${L}_{c}=50 \mathrm{\mu m}$ (see text for details).

We can now assess the relative importance of the three dimensionless groups in the phase map of Fig. 6 for a range of inhaled aerosol sizes known to reach the acinar regions^4,5^, and a range of net charges *n*(e) representative of various inhaler devices^8^. The distinctive zones in the phase map underline areas where one group dominates over the others while the delineation between zones identifies where two groups are equal in an order of magnitude sense. We emphasize that dimensional analysis supports a comprehensive assessment of aerosol transport in our in vitro experiments in contrast to directly predicting physiological acinar deposition outcomes^66^. Namely, we only consider the transport determinants for particles that have effectively entered the acinar regions^5,50^, thereby omitting entirely the fate of deposited aerosols screened along the inhalation route spanning mouth to distal airways^67^.

With such limitations in mind, we first revisit our experimental observations (Figs. 3, 4 and 5) for the case of charged aerosols. In the absence of knowing the exact number of charges on the PSL particles, Fig. 6a underlines how the Inc group largely dominates deposition when aerosols hold charges of several hundred electrons as estimated in our specific exposure setup^48^. With further increases in charge (*n*(e) > 10^3^), Inc will also dominate deposition for aerosols larger than those explored in our experiments (*d*_*p*_ > 1.1 µm). We note that each dimensionless parameter depends on a characteristic velocity scale *U*_*c*_, where we have concentrated on local alveolar transport phenomena. Here, we have chosen the mean velocity in the recirculating cavities (*U*_*c*_ = 3 mm/s) following CFD simulations (Fig. 1d). Since all three mechanisms are inversely proportional to *U*_*c*_ the interplay between the dimensionless groups is unaffected by either slower or faster airflows in the realm of low Re anticipated in the acinar regions.

Concurrently, Inc is inversely proportional to the square of the characteristic length *L*_*c*_. Here, we defined *L*_*c*_ = *D*_*a*_/4 = 50 µm which corresponds to the distance halfway from the alveolar wall to the vortex center but also equals our septal length *L*_*s*_. We specifically opted for a length scale based on the recirculating alveolar flow topology (Fig. 1e) to capture typical interplays between deposition mechanisms within alveoli rather than limiting ourselves only to very short distances in the vicinity of the lumen.

We note that in our experiments the Pe number holds relatively large values when considering the mean (channel) velocity, e.g. Pe ~ 550 for ${d}_{p}=0.2$ µm. Such values underscore the significance of advection in the transport of aerosols and their trajectory. Yet, the exact fate of such aerosols depends on a superposition between deterministic advection and stochastic Brownian motion. In this study, we foremost explored particle deposition, where advection plays a role in transporting particles but does not directly lead to their deposition on the wall. Within the scope of this work, our primary objective was thus to investigate the intricate interplay between H, Pe, and Inc on deposition outcomes. These non-dimensional numbers are defined with respect to the advection term ($\propto {U}_{c}^{-1})$. By comparing them as ratios, we effectively eliminate the fixed-velocity advection term, enabling us to focus on their mutual relationships.

In Fig. 6b, our phase map underlines how for sub-micron particles (e.g. < 170–200 nm), there appears to be a rather sharp cutoff when particles with charges > 100 e are dominated by electrostatic effects, whereas for those with < 100 e diffusional mechanisms will largely prevail. In contrast as particles become larger (i.e. micron-sized aerosols), the coupling between size and charge becomes stronger as highlighted in the interplay between Inc and H. Regardless, our choice for the length *L*_*c*_ does not fundamentally alter the existence of the three distinctive zones in the phase map (Fig. 6), underscoring the importance of including Inc to characterize tangible electrostatic effects.

## Conclusions

In the present paper, we have experimentally explored electrostatic charge effects on aerosol deposition in a canonical in vitro model of an alveolated microchannel. Our results point to the highly important role of electrostatic forces for aerosols entering the acinar depths. Notably, our data further support why septal deposition is anticipated to prevail at the cost of lower alveolar cavity deposition given the anatomical arrangement of the pulmonary acinus. In conjunction with our results from dimensional analysis, we may conclude that while neglecting the role of electrostatic charge in the acinar regions may be relevant for nebulizers and spacers, this will not be necessarily true for aerosols smaller than 2 μm produced by commercially-available inhalers such as in the case of DPI and MDI (as inferred from Fig. 6a)^8^. Undoubtedly, further studies are still needed to deepen our understanding of the interplay of electrostatic effects in more faithful acinar environments (e.g., alveolar wall movements, high relative humidity, etc.). Yet, looking back at the International Commission on Radiological Protection (ICRP)^68^, the present work, and those that lie ahead, give reason to advocate for revised guidelines in understanding comprehensively pulmonary deposition in the distal airways and acinar regions in the presence of electrostatic charge.

### Supplementary Information

Supplementary Information.

Supplementary Video 1.

## Data Availability

Additional details can be found in the Supplementary Material (SM) accompanying this paper.
