# Supplementary material for: Exploring the role of electrostatic deposition on inhaled aerosols in alveolated microchannels
Source: Sci Rep. 2023 Dec 27;13:23069. doi: 10.1038/s41598-023-49946-w (PMC10754925; doi:10.1038/s41598-023-49946-w)
Supplement: Supplementary file 1 — Supplementary Information. [file 41598_2023_49946_MOESM1_ESM.docx]

*Supplementary Material*

Exploring the role of electrostatic deposition on inhaled aerosols in alveolated microchannels

Ron Bessler^1^, Saurabh Bhardwaj^2^, Daniel Malka^1^, Rami Fishler^1^ and Josué Sznitman^1,^^[[1]](#footnote-2)^

^1^ Department of Biomedical Engineering, Technion – Israel Institute of Technology, Haifa, Israel

^2^ Department of Biomedical Engineering, Pennsylvania State University, State College, PA, USA

# Derivation of Non-dimensional Inc

Followed by Newton’s 2^nd^ law:

|  | $\frac{d\vec{P}_{p}}{dt}=\vec{F}_{gravity}+\vec{F}_{drag}+\vec{F}_{electostatic}^{Inc}+\vec{F}_{Brownian}$ | (1) |
| --- | --- | --- |

For this discussion, to simplify the derivation, we will not elaborate on the well-known Brownian motion force. In the absence of hygroscopic growth:

|  | $\frac{d\vec{P}_{p}}{dt}\approx m_{p}\left( \frac{d\vec{u}_{p}}{dt} \right)$ | (2) |
| --- | --- | --- |

Gravity force is:

|  | $\vec{F}_{gravity}=-m_{p}g \hat{Z}$ | (3) |
| --- | --- | --- |

The drag force is:

|  | $\vec{F}_{drag}=\frac{1}{2}\rho_{f}u_{rel}^{2}\left( \frac{\pi d_{p}^{2}}{4} \right)C_{d} \left( -\hat{z} \right)$ | (4) |
| --- | --- | --- |

Where $\vec{u}_{rel}= \vec{u}_{p}-\vec{u}_{f}$ is the relative velocity and $C_{d}$ is the drag coefficient. For a small sphere where $Re_{p}=\frac{\rho_{f}d_{p} u_{rel}}{\mu_{f}}\approx0.01\ll1$
the drag coefficient is $C_{d}\approx\frac{1}{C_{c}}\frac{24}{\mathrm{Re}_{p}}$ (also known as Stokes drag) where $C_{c}$ is the Cunningham slip correction factor:

|  | $C_{c}=1+2K_{n}\left( A_{1}+A_{2}e^{-{A_{3}K}_{n}} \right)$ | (5) |
| --- | --- | --- |

Where $A_{1}=1.257, A_{2}=0.400, A_{3}=0.275$ based on experiments [16]. $K_{n}$ is Knudsen dimensionless number, determine whether continuum mechanics or statistical mechanics formulation of fluid dynamics should be used for the given problem. It is defined as the ratio of the molecular mean free path length $\lambda(T)$ to a representative physical length scale ($d_{p})$:

|  | $K_{n}=\frac{\lambda\left( T \right)}{d_{p}}$ | (6) |
| --- | --- | --- |

The mean free path $\lambda(T)$ is the average distance over which a moving molecule, which consists of the hosted fluid, free to move, does not collide with each other. This property depends strongly on the temperature: for room temperature ($T={22}^{0}[C])$ or lungs ($T={36.6}^{0}[C]$ ).

Then the drag force is corrected to:

|  | $\vec{F}_{drag}=-\frac{3\pi d_{p}\mu_{f}}{C_{c}} u_{rel}\left( -\hat{z} \right)$ | (7) |
| --- | --- | --- |

The electrostatic force between two charged particles is:

|  | $\vert\vec{F}_{electostatic}\vert=\frac{q_{1}q_{2}}{4\pi\varepsilon}\frac{1}{x^{2}}$ | (8) |
| --- | --- | --- |

In the particle-tissue interaction, the aerosol electric field causes the charged molecules on the neutralized tissue to reorient themselves (i.e., this process is known as the dielectric effect) in a way that keeps the electric field within the tissue zero, as in a conductor. Since the dimension of the tissue is much larger compared to the particle, this process can be equivalently viewed as if there were a mirror distance charge with an equal magnitude, but an opposite polarity induced within the tissue (${|q}_{1}|=\left| q_{2} \right|=q$), see Fig 1 in the paper. Since both the induced and real particles must be oppositely charged, regardless of particle polarity, there is always an attractive force between the particle and the tissue wall which can potentially enhance deposition.

The distance $r=2x$, where $x$ is the distance from the infinite tissue wall. Additionally, with the approximation that the permittivity of the tissue is similar to that of water $\varepsilon_{water}\sim80\varepsilon_{0}$ $\frac{C^{2}}{Nm^{2}}$ which is larger than that of the air $\varepsilon_{air}\sim1\varepsilon_{0} \frac{C^{2}}{Nm^{2}},$
where $\varepsilon_{0}$ is the dielectric constant of vacuum $8.85\times{10}^{-12} \left( \frac{F}{m} \right)$.
We can estimate that:

|  | $\vec{F}_{electostatic}^{Inc}=\frac{q^{2}}{x^{2}16\pi\varepsilon_{0}}\hat{r}$ | (9) |
| --- | --- | --- |

It is more convenient to use the Coulomb constant: $K_{e}=1/4\pi\varepsilon_{0}$

|  | $\vec{F}_{electostatic}^{Inc}=\frac{K_{e}q^{2}}{4x^{2}}\hat{r}$ | (10) |
| --- | --- | --- |

Substituting all the forces:

|  | $m_{p}\left( \frac{d\vec{u}_{p}}{dt} \right)=-m_{p}g \hat{Z}-\frac{3\pi d_{p}\mu_{f}}{C_{c}}u_{rel} \hat{z}+\frac{K_{e}q^{2}}{4x^{2}}\hat{r}$ | (11) |
| --- | --- | --- |

Substituting the mass for sphere particle with unified density:

|  | $m_{p}=\rho_{p}\frac{\pi d_{p}^{3}}{6}$ | (12) |
| --- | --- | --- |

And normalize the ODE:

|  | $\left( \frac{d\vec{u}_{p}}{dt} \right)=-g \hat{Z}-\underset{1\backslash\tau_{p}}{\underbrace{\frac{18\mu_{f}}{{{\rho_{p}d}_{p}^{2}C}_{c}}}}u_{rel} \hat{z}+\frac{3}{\rho_{p}d_{p}^{3}}\frac{K_{e}q^{2}}{2x^{2}}\hat{r}$ | (13) |
| --- | --- | --- |

Defining the particle relaxation time as:

|  | $\tau_{p}=\frac{C_{c}\rho_{p}d_{p}^{2}}{18\mu_{f}}$ | (14) |
| --- | --- | --- |

It is also common to define the Mechanical Mobility B

|  | $B=\frac{\tau_{p}}{m_{p}} =\frac{\left( \frac{\rho_{p}d_{p}^{2}C_{c}}{18\mu_{f}} \right)}{\left( \frac{{\rho_{p}\pi d}_{p}^{3}}{6} \right)}=\frac{C_{c}}{3\pi d_{p}\mu_{f}}$ | (15) |
| --- | --- | --- |

We will have:

|  | $\frac{d\vec{u}_{p}}{dt}=g \left( -\hat{Z} \right)+\frac{u_{rel}}{\tau_{p}} (-\hat{z})+\frac{3}{\rho_{p}d_{p}^{3}}\frac{K_{e}q^{2}}{2x^{2}}\hat{r}$ | (16) |
| --- | --- | --- |

Substitute the non-dimensional numbers as follows:

$$u_{p}^{'}=\frac{u_{p}}{U_{c}} u_{f}^{'}=\frac{u_{f}}{U_{c}} u_{rel}^{'}=u_{p}^{'}-u_{f}^{'}$$

$t^{'}=\frac{t}{(L_{c}\backslash U_{0})}$ $x'=\frac{x}{L_{c}}$ $g^{'}=\frac{g}{g_{0}}$

We will have

|  | $\underset{\mathrm{Stk}}{\underbrace{\frac{\tau_{p}U_{c}}{L_{c}}}} \frac{d{\vec{u}^{'}}_{p}}{dt'}=\underset{H}{\underbrace{\frac{g_{0}\tau_{p}}{U_{c}}}}g^{'} \left( -\hat{Z} \right)+{u^{'}}_{rel}\left( -\hat{z} \right)+\underset{\mathrm{Inc}}{\underbrace{\frac{K_{E}B}{{4U}_{c}} \left( \frac{q}{L_{c}x^{'}} \right)^{2}}}\hat{r}$ | (17) |
| --- | --- | --- |

where:

$\frac{\tau_{p}U_{c}}{L_{c}}=\mathrm{Stk}$ $\frac{g_{0}\tau_{p}}{U_{c}}=H$ $\frac{K_{E}B}{{4U}_{c}} \left( \frac{q}{L_{c}x^{'}} \right)^{2}=\mathrm{Inc}$

For shorts, we can write:

|  | $L_{c}^{e}=L_{c}x^{'}$ | (18) |
| --- | --- | --- |

Hence, the non-dimensional governing equation is [17]:

|  | $\mathrm{Stk}\frac{d\vec{u}_{p}'}{dt'}=H g^{'}\left( -\hat{Z} \right)-u_{rel}^{'} (-\hat{z})+\mathrm{Inc} (\hat{r})$ | (19) |
| --- | --- | --- |

We can define the ratio between the groups:

|  | $\frac{\mathrm{Inc}}{H}=\frac{3}{8\pi^{2}d_{p}^{3}\rho_{p}\varepsilon_{0}g}\left( \frac{q}{L_{c}^{e}} \right)^{2}$ | (20) |
| --- | --- | --- |
|  | $\frac{\mathrm{Inc}}{\mathrm{Stk}}=\frac{3{L_{c}K}_{e}}{2\rho_{p}\pi d_{p}^{3}U_{c}^{2}}\left( \frac{q}{L_{c}^{e}} \right)^{2}$ | (21) |

# Diffusion - The Inverse Pe Number:

The Péclet number (Pe) is a dimensionless number defined as the ratio of the rate of advection versus the flow rate of diffusion:

|  | $\mathrm{Pe}=\frac{advection rate}{diffusion rate}$ | (22) |
| --- | --- | --- |

Instead of a rates scale, we can use the inverse parallel concept time scale $\left( f=\frac{1}{T} \right)$:

|  | $\mathrm{Pe}=\frac{(1/\tau_{\mathrm{advec}})}{(1/\tau_{\mathrm{diff}})}=\frac{\tau_{\mathrm{diff}}}{\tau_{\mathrm{advec}}}$ | (23) |
| --- | --- | --- |

The advection time scale is

|  | $\tau_{\mathrm{advec}}\sim L_{c}^{\mathrm{advec}}/U_{c}$ | (24) |
| --- | --- | --- |

The characteristic time scale can be derived by the characteristic diffusion length based on the root-mean-squared (RMS) displacement diffusion:

|  | $<x^{2}> =kD_{diff}t$ | (25) |
| --- | --- | --- |

Where $k$ is the constant dimension number, for $k_{1D}=2, k_{2D}=4$and $k_{3D}=6$.

$D$ is the spherical particle diffusion coefficient given by the Stokes-Einstein relation,

|  | $D=K_{B}TB$ | (26) |
| --- | --- | --- |

$K_{B}$is Boltzmann's constant and *T* is the absolute temperature (in Kelvin), $B$ is the mechanical mobility.

|  | $B=\frac{\tau_{p}}{m_{p}}=\frac{\left( \frac{\rho_{p}d_{p}^{2}C_{c}}{18\mu_{f}} \right)}{\left( \frac{{\rho_{p}\pi d}_{p}^{3}}{6} \right)}=\frac{C_{c}}{3\pi d_{p}\mu_{f}}$ | (27) |
| --- | --- | --- |

Hence, we can deduce $\tau_{\mathrm{diff}}$ as

|  | $\tau_{\mathrm{diff}} \sim\frac{L_{c}^{2}}{D_{diff}}$ | (28) |
| --- | --- | --- |

Back to the basic definition we get:

|  | $\mathrm{Pe}=\frac{\tau_{\mathrm{diff}}}{\tau_{\mathrm{advec}}}=\frac{\left( {L^{\mathrm{diff}}}_{c}^{2}/D_{diff} \right)}{( L_{c}^{\mathrm{advec}}/U_{c})}=\frac{{L_{c}^{diff}}^{2}}{L_{c}^{advec}}\frac{U_{c}}{D_{diff}}$ | (29) |
| --- | --- | --- |

Assuming $L_{c}^{\mathrm{advec}}=L_{c}^{\mathrm{diff}}=L_{c}$ we get

|  | $\mathrm{Pe}=\frac{L_{c}U_{c}}{D_{diff}}$ | (30) |
| --- | --- | --- |

We are interested in the inverse Peclet number since we want to find under which condition the diffusion is dominant.

|  | $\mathrm{Pe}^{-1}=\frac{D_{diff}}{L_{c}U_{c}}$ | (31) |
| --- | --- | --- |

And for the difference in distance

|  | $\mathrm{Pe}^{-1}=\frac{D_{diff}}{U_{c}}\frac{L_{c}^{\mathrm{advec}}}{{L_{c}^{\mathrm{diff}}}^{2}}$ | (32) |
| --- | --- | --- |

In comparison to electrostatic, Induced Charge (Inc) (where: $L_{c}^{\mathrm{advec}}=L_{c}^{\mathrm{diff}}=L_{c})$

|  | $\frac{\mathrm{Inc}}{\mathrm{Pe}^{-1}}=\frac{{K_{e}q}^{2}L_{C}}{4K_{B}T}$ | (33) |
| --- | --- | --- |

## Electrostatic versus Diffusional and Gravitational Mechanisms

To find an approximation for electrostatic charge $q$ to play a significant role compared to the other well-accepted mechanism (gravitational sedimentation and Brownian diffusion) in the acinar region, we present the following analysis according to Newton’s second law:

|  | $\Sigma F=m\frac{d^{2}x}{dt^{2}}$ | (34) |
| --- | --- | --- |

Using non-dimensional analyses for the acinar region we find that STK$=\frac{\tau_{p}U}{L_{c}}$ << H, Inc,1/Pe, hence we can neglect the inertia term, substituting electrostatic force and drag:

$$-\frac{kq^{2}}{\left( 2x \right)^{2}}-\frac{1}{B}\dot{x}\approx0$$

$$\frac{kq^{2}}{4}\frac{1}{x^{2}}=-\frac{1}{B}\frac{dx}{dt}$$

$$\frac{kq^{2}B}{4}dt=-x^{2} dx$$

$$\int_{0}^{t_{f}} \frac{kq^{2}B}{4}dt=-\int_{x\left( 0 \right)=x_{0}}^{x\left( t_{f} \right)=0} x^{2} dx$$

$$\frac{kq^{2}B}{4}t_{f}=+\frac{x_{0}^{3}}{3}$$

And we get the connection between distance and time:

|  | $x_{0}^{3}=\frac{3kq^{2}B}{4}t_{f}$ | (35) |
| --- | --- | --- |

This was previously shown in [2]. Now we would find an appropriate time characteristic for each mechanism.

1. diffusion comparison:

The characteristic time scale can be derived by the characteristic diffusion length based on the root-mean-squared (RMS) displacement for 2D diffusion:

|  | $<x^{2}> =4Dt$ | (36) |
| --- | --- | --- |

|  | $t_{f} \sim\frac{x_{0}^{2}}{4D}$ | (37) |
| --- | --- | --- |

where $D$ is the spherical particle diffusion coefficient given by the Stokes-Einstein relation,

|  | $D=K_{B}TB$ | (38) |
| --- | --- | --- |

$K_{B}$is Boltzmann's constant and *T* is the absolute temperature (in Kelvin). Substituting *D* and we will get:

$$t_{f} \sim\frac{x_{0}^{2}}{4K_{B}T B}$$

Substitute $t_{f}$ from eq (35) will yield:

$$x_{0}^{3}=\frac{3kq^{2}B}{4}*\left( \frac{x_{0}^{2}}{4 K_{B}T B} \right)$$

|  | $x_{0}=\frac{3k}{16K_{B}T}q^{2}$ | (39) |
| --- | --- | --- |

reorganize for the charge require:

|  | $q_{d}=\sqrt{\frac{16K_{B}T x_{0}}{3k}}$ | (40) |
| --- | --- | --- |

1. Gravitational Sedimentation

For gravitational sedimentation comparison, the terminal settling velocity was used.

|  | $t_{f} \sim\frac{x_{0}}{v_{set}}=\frac{x_{0}}{\tau_{p}g}$ | (41) |
| --- | --- | --- |

Substitute $t_{f}$ of our previous equation within eq (35) :

$$x_{0}^{3}=\frac{3kq^{2}B}{4}\frac{x_{0}}{\tau_{p}g}$$

Recall that $\tau_{p}=mB$

|  | $x_{0}^{2}=\frac{3kq^{2}}{4mg}$ | (42) |
| --- | --- | --- |

reorganize for the charge require:

|  | $q_{s}=2x_{0}\sqrt{\frac{mg}{3k}}$ | (43) |
| --- | --- | --- |

We calculated the length achieved within 1 sec by the conventional mechanisms: Sedimentation (PSL particles $\rho_{p}=1050 kg/m^{3}$) and diffusion (at Room temperature). For a more conservative approach, we weaken the electrostatic force, we present the distance from the wall to be as: $x_{0}=L_{c}+l_{mechanism at 1sec}$ Where $L_{c}$ is the characteristic length. For our problem, we have chosen this distance to be the mid-distance for the particle to be away from the alveoli wall $L_{c}=\frac{r_{a}}{2}=50 \mu m$. The results summarized in Table 1.

## Table 1

Characteristic distance (µm) and equivalent charge (e) resulting from settling and diffusional transport mechanisms estimated for the particle sizes investigated here.

| Particle  Diameter  $d_{p}$($\mu m)$ | Settling | | Diffusion | |
| --- | --- | --- | --- | --- |
|  | Characteristic distance in 1 sec $\left( \mu m \right)$  $l_{s}=t*v_{set}$ | Equivalent  charge  $q_{s}(e)$ | Characteristic distance in 1 sec $\left( \mu m \right)$  $l_{d}=\sqrt{4Dt}$ | Equivalent charge  $q_{d}(e)$ |
| $0.2$ | $2.34$ | $27$ | $29.90$ | $88$ |
| $0.5$ | $10.62$ | $120$ | $16.11$ | $80$ |
| $1.1$ | $44.33$ | $608$ | $10.08$ | $76$ |

# Electrostatic Relaxation Time

The ability of tissue charges to adapt their orientation in response to an external electric field, such as one generated by a charged airborne particle in the lumen, is a consequence of solving the current continuity equation:

|  | $\vec{\nabla}\cdot\vec{j}+\frac{\partial\rho_{e}}{\partial t}=0$ | (44) |
| --- | --- | --- |

Where $\vec{J} \left( \frac{e}{{s\cdot m}^{2}} \right)$ is the current density and $\rho_{e}\left( \frac{e}{m^{3}} \right)$ is the volume charge density.

Using Guess law, and Ohms law where $\sigma$ is the tissue conductivity, we can simplify the divergence of the current density $\vec{\nabla}\cdot\vec{j}$

|  | $\underset{Guess law}{\underbrace{\vec{\nabla}\cdot\vec{E}=\frac{\rho_{e}}{\varepsilon}}}$ | (45) |
| --- | --- | --- |

|  | $\underset{Ohms law}{\underbrace{\sigma\vec{E}=\vec{J}}}$ | (46) |
| --- | --- | --- |

$$\to\vec{\nabla}\cdot\left( \frac{1}{\sigma} \vec{J} \right)=\frac{\rho_{e}}{\varepsilon} \to\vec{\nabla}\cdot\vec{J}=\frac{\sigma}{\varepsilon}\rho_{e}$$

Substitute back into current continuity equation, wea are facing a time ODE for $\rho_{e}(t)$:

|  | $\frac{\sigma}{\varepsilon}\rho_{e}+\frac{\partial\rho_{e}}{\partial t}=0$ | (47) |
| --- | --- | --- |

Its solution is:

|  | $\rho_{e}(t)=\rho_{0}e^{-t/\tau_{e}}$ | (48) |
| --- | --- | --- |

Where the $\tau_{e}$is the electrical time constant define as $\tau_{e}=\varepsilon/\sigma$ and $\rho_{0}$ is a charge density constant. Units check: $\tau_{e}=\frac{\left[ \frac{F}{m} \right]}{\left[ \frac{1}{\Omega m} \right]}=\frac{\left[ F \right]}{\left[ 1/\Omega\right]}=\frac{\left[ F \right]}{\left[ F/s \right]}=[s]$

In Table 2 we summarize the different electrical properties which include conductivity, dielectric constant, and the electrical time constant, where $\varepsilon_{0}$ is the dielectric constant of vacuum $8.85\times{10}^{-12} \left( \frac{F}{m} \right)$. To ensure that the charge density $\rho_{e}(t)$ within the microchannel aligns with the tissue's behavior, it's essential to establish a similar relationship between the electrical time constant $\tau_{e}$ and other time constants associated with the flow, denoted as $\tau_{\mathrm{flow}}$. There exist various alternative time scales that characterize flow, including the breathing rate ($\tau_{\mathrm{Breathing}}=4 [s]$), the advective time scale of the airway ($\tau_{\mathrm{advective}}=\frac{L_{c}}{U_{ave}}=\frac{50\mu m}{0.1 m/s}={5\times10}^{-4} [s]$), and even the particle relaxation time ($\tau_{p}=\rho_{p}d_{p}^{2}/18\mu_{f}\sim{10}^{-6} [s]$). However, regardless of the specific choice among these alternatives, it's important to note that electrostatic effects within the tissue occur on a much shorter timescale compared to the flow ($\tau_{e}\ll\tau_{flow}$). Therefore, any selection that adheres to this relationship will be suitable for faithfully replicating the anticipated electrostatic effects within the lung, as we aim to achieve in our research. Conductive materials, including metals, are viable choices for this purpose [3]. However, in our research, we selected ITO due to its transparency feature, which ensures minimal interference with our particle measurements.

## Neutralize charge distributions – Boltzmann distribution

The minimum charge aerosols can achieve is zero, but this is rarely achieved because of the random thermal collisions with the ions found in the fluid. Even a high amount of charged aerosols will slowly lose their charge to an equilibrium charge state known as Boltzmann charge equilibrium.

The fraction of particles with $n$ elementary charges is given by [3] :

| $f_{n}=\frac{\exp\left[ g(i) \right]}{\sum_{i=-\infty}^{i=+\infty} \left\{ \exp\left[ g\left( i \right) \right] \right\}}$ | (49) |
| --- | --- |

Where the function with the running index $i$, is dependent on the size of the particle and the temperature of the environment, and it is given by

| $g\left( i,T,d_{p} \right)=\frac{k\left( ie \right)^{2}}{d_{p}K_{B}T}$ | (50) |
| --- | --- |

Mostly for particles larger than ${0.05 [\mu m]<d}_{p}$, it is well common to approximate the exponential ratio into easier-to-use form as:

| $f_{n}\approx\sqrt{\frac{g\left( n \right)}{\pi i^{2}}}*\exp\left[ -g(n) \right]$ | (51) |
| --- | --- |

The probability distribution function (PSD) is symmetrical for the positive and negative signs but highly depends on the size of the particle. In Fig 2 we plot the PDF versus the charge size used in our experiments. It is important to bear in mind that even though the distribution is symmetrical for the charge sign, the average magnitude of the charge is non-zero and differs with size, see Table 3. The calculation for the average charge is based on experimental results with an accuracy of $\pm5\%$ given by the following equation.

| $\bar{n}\approx2.37\sqrt{d_{p}[\mu m]}$ | (52) |
| --- | --- |

Note: the real charge can be only a whole multiplication of the charge unit [e] and this is only a mathematical deduction to emphasize that charge is non-zero for the majority aerosol cloud even for the Boltzmann distribution. This concept intensified with the particle size according to equation (52). As we can see from the graph, the neutralizer is suppressing the charge down to several electrons, which is significantly smaller by two orders of magnitude than expected when bypassing it.

## Calculation of the concentration ratio

The differences between septal and alveolar deposition were evaluated using the particle deposition density (or concentration) defined as *C=*(DF)/*A*; We were specifically interested in evaluating the ratio *C_s_*/*C_a_* to assess the relative importance of septal deposition to an alveolar deposition where *C_s_* and *C_a_* are the septal and alveolar deposition densities respectively.

This ratio is inversely proportional to the area of each domain followed by

| $\frac{C_{s}}{C_{a}}=\frac{\left( \frac{{DF}_{s}}{A_{s}} \right)}{\left( \frac{{DF}_{a}}{A_{a}} \right)}=\frac{{DF}_{s}}{{DF}_{a}}\left( \frac{A_{a}}{A_{s}} \right)$ | (53) |
| --- | --- |

Whereas *A_s_* and *A_a_* correspond to the total septal and alveolar surface areas, respectively. The rectangular area of a single septum is $A_{s}=bL_{s}$ where *b* is the depth (y coordinates) and $l$ is the length (x coordinates). Based on the findings from our inverse microscope bright field mode, See Fig 3, each diced alveoli curvature is approximately 8 times longer than the septa size $L_{s}$. Since both areas have the same depth in the y-axis direction, we can calculate the ratio of the areas:

| $\frac{A_{a}}{A_{s}}=\frac{b(8L_{s})}{bL_{s}}=8$ | (54) |
| --- | --- |

On top of that, we recall that our microchannel contains 6 alveolar cavities and 5 septal spacings.

$$\frac{C_{s}}{C_{a}}=\frac{6}{5}\frac{{DF}_{s}}{{DF}_{a}}\left( \frac{A_{a}}{A_{s}} \right)\approx\frac{6}{5}\frac{{DF}_{s}}{{DF}_{a}}8=9.6\frac{{DF}_{s}}{{DF}_{a}}$$

| $\frac{C_{s}}{C_{a}}=9.6\frac{{DF}_{s}}{{DF}_{a}}$ | (55) |
| --- | --- |

Hence, the factor of the transition between the ratio of the Deposition Fraction into the Concertation Ratio differs by the constant 9.6 which accounts for the area difference of the total areas.

## Deposition within the Cavities

In Fig 4, we plotted the summary deposition fraction (DF) within each cavity. The horizontal axis is the normalized distance $x/D_{a}$ where $D_{a}$ is the alveoli length (diameter). We present DF according to particle size in the rows. The group i.e. neutralized (light gray histograms) versus charged (dark gray histograms) presented in the columns. The histograms are normalized according to the total number of deposited particles.

# References

[1] W. H. Finlay, *The Mechanics of Inhaled Pharmaceutical Aerosols: An Introduction*. Elsevier Science, 2001.

[2] V. Prodi and A. Mularoni, “Electrostatic Lung Deposition Experiments with Human and Animals,” *Ann. Occup. Hyg.*, vol. 29, no. 2, pp. 229–240, Apr. 1985, doi: 10.1093/annhyg/29.2.229.

[3] W. C. Hinds and Y. Zhu, “Electrical Properties,” in *Aerosol Technology: Properties, Behavior, and Measurement of Airborne Particles*, Wiley, 2022, pp. 277–304.

# Acknowledgments

The authors thank Bar Kalifa, Morane Elbaz, Gal Yanuka, and Nitsan Dahan (Technion) as well as Dr. Orna Ternyak (Russell Berrie Nanotechnology Institute, Technion) for technical support. This research was supported by the Israel Science Foundation (Grant no. 1840/21).

# Conflict of interest

The authors declare having no conflict of interest.

# Figure Captions

Figure 1

Schematic drawing of the attractive electrostatic force between a charged particle and its image charge in proximity to an electrically conductive wall surface

Figure 2

Boltzmann charge probability distribution function (PSD) for our 3 particle sizes. The three particles’ sizes are arranged according to brightness, with the lightest gray being the smallest and the darker gray being the largest.

Figure 3

Bright-field mode image, captured using an inverted microscope, reveals that our septal distance $L_{s}$ is approximately 8 times shorter than the alveolar curvature length.

Figure 4

Histogram with the inner resolution on the deposition fraction of the cavity for each particle size. The horizontal axis represents a normalized distance, each bin represents an equal distance of 8.3 μm.

# Figures

## Fig 1:

$$\mathrm{Conductor}$$

$$x$$

$$x$$

$$F_{elec}$$

$$\hat{x}$$

$$F_{drag}$$

+

-

$$\mathrm{Air}$$

## Fig 2:


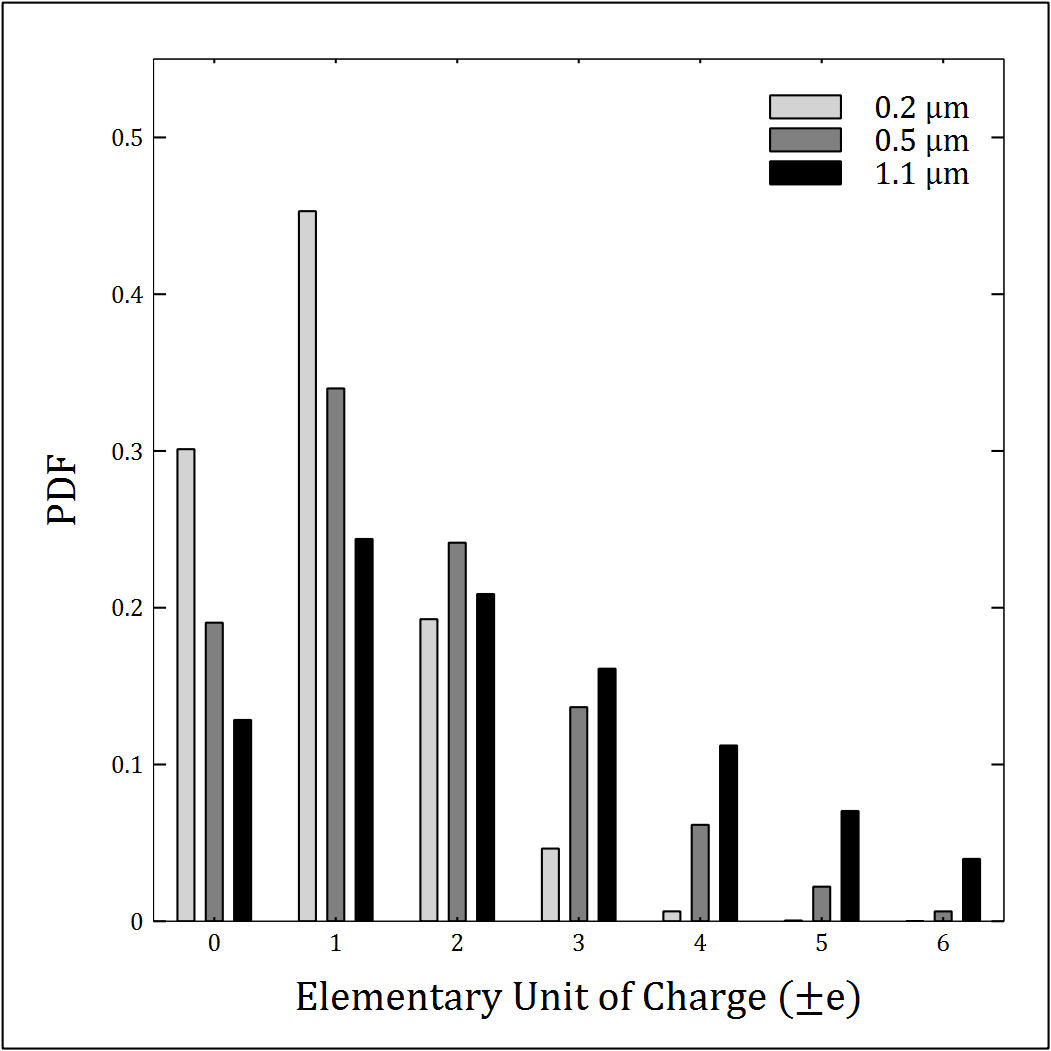


## Fig 3:


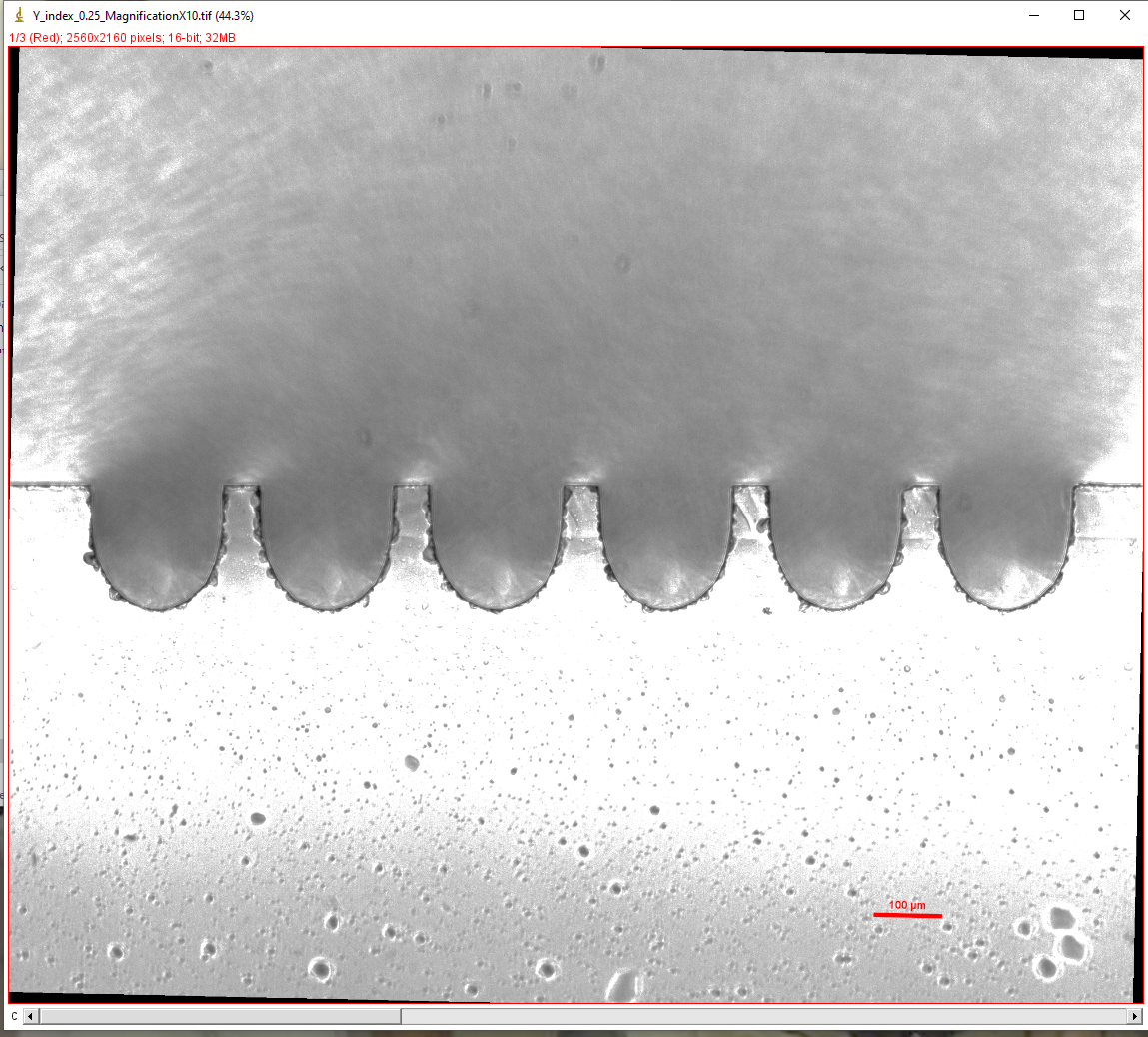

$$L_{s}$$

$$8L_{s}$$

## Fig 4.


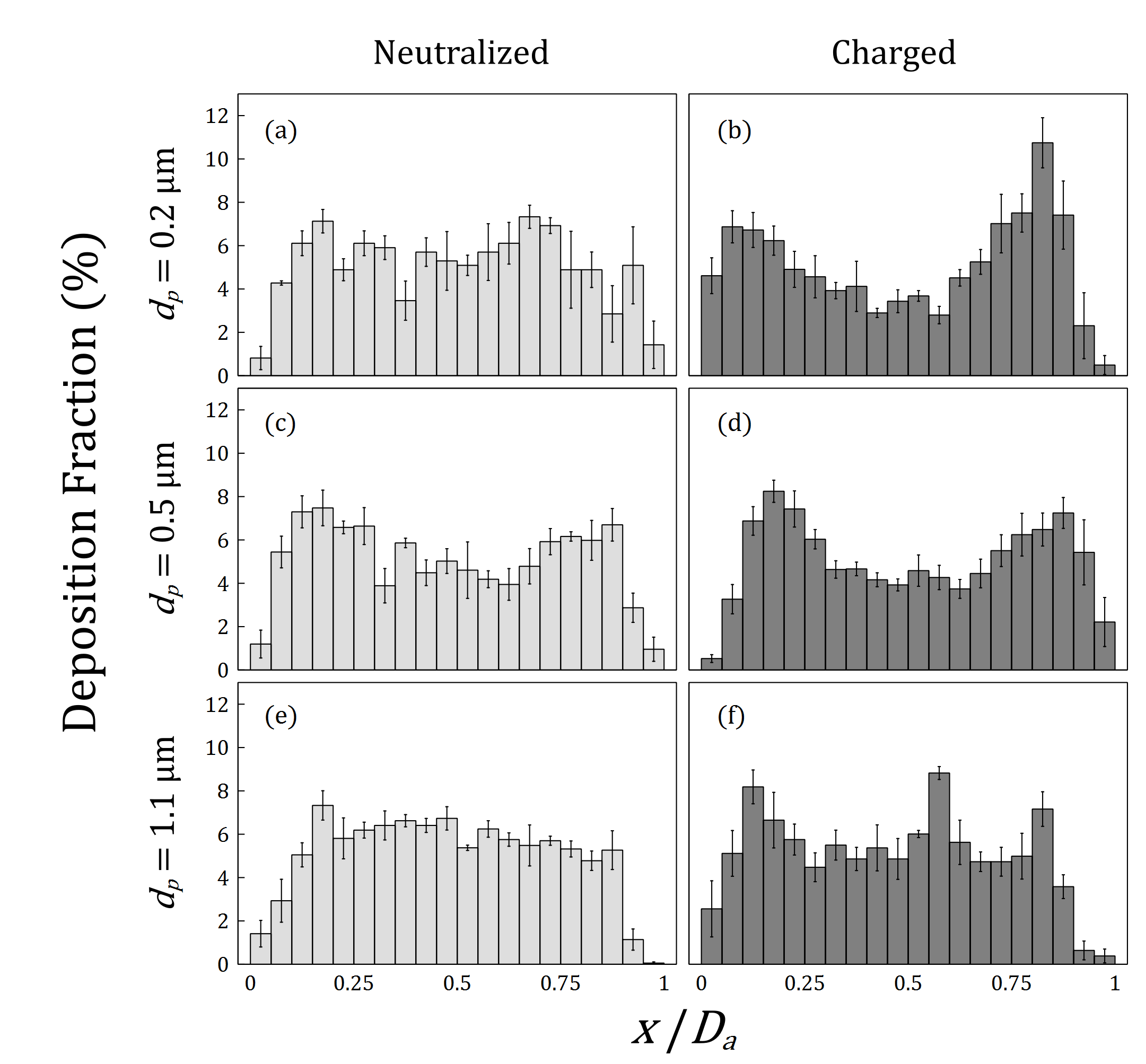


# Tables

## Table 1 - Characteristics of distances and charge

Characteristic distance (µm) and equivalent charge (e) resulting from settling and diffusional transport mechanisms estimated for the particle sizes investigated here.

| Particle Diameter ($\mu m)$ | Settling length in 1 sec $\left( \mu m \right),$  $l_{s}=t*v_{set}$ | Equivalent Performance $q_{s}(e)$ | Diffusion length in 1 sec $\left( \mu m \right),$  $l_{d}=\sqrt{4Dt}$ | Equivalent Performance $q_{d}(e)$ |
| --- | --- | --- | --- | --- |
| $0.2$ | $2.34$ | $27$ | $29.90$ | $88$ |
| $0.5$ | $10.62$ | $120$ | $16.11$ | $80$ |
| $1.1$ | $44.33$ | $608$ | $10.08$ | $76$ |

## Table 2 – Average charge

Electrical properties of glass, water and ITO

| Material | Conductivity  $\sigma\left[ S/m \right]$ | Dielectric Constant $\varepsilon[F/m]=\varepsilon_{r}\varepsilon_{0}$ | Electrical time constant  $\tau_{e}[s]=\frac{\varepsilon}{\sigma}$ |
| --- | --- | --- | --- |
| Glass | $\sigma_{\mathrm{Glass}} \sim{10}^{-11}$ | $\varepsilon_{\mathrm{Glass}} \sim5 \varepsilon_{0}$ | $44.25 [s] \sim1[min]$ |
| $H_{2}O$ | $\sigma_{H_{2}O} \sim1$ | $\varepsilon_{H_{2}O} \sim80 \varepsilon_{0}$ | $\sim7\times{10}^{-10}[s]$ |
| ITO | $\sigma_{\mathrm{ITO}}\sim{10}^{4}$ | $\varepsilon_{\mathrm{ITO}} \sim3 \varepsilon_{0}$ | $\sim{10}^{-15}[s]$ |

## Table 3 – Average charge

Average charge versus the particle diameter according to Boltzmann distribution.

| $\bar{n}$ (e) | $d_{p}(\mu m)$ |
| --- | --- |
| 1.06 | 0.2 |
| 1.67 | 0.5 |
| 2.48 | 1.1 |

1. ^*^Corresponding author: sznitman@bm.technion.ac.il [↑](#footnote-ref-2)
